# Supplementary material for: Randomised, placebo-controlled, phase 3 trial of the effect of the omega-3 polyunsaturated fatty acid eicosapentaenoic acid (EPA) on colorectal cancer recurrence and survival after surgery for resectable liver metastases: EPA for Metastasis Trial 2 (EMT2) study protocol
Source: BMJ Open. 2023 Nov 29;13(11):e077427. doi: 10.1136/bmjopen-2023-077427 (PMC10689403; doi:10.1136/bmjopen-2023-077427)
Supplement: Supplementary data [file bmjopen-2023-077427supp002.pdf]

		Visit 1 (randomisation)	Fortnightly pre-op telephone call	Visit 2: Surgery	Visit 3 (6 months)*	Visit 4 (12 months)	Visit 5 (18 months)	Visit 6 (24 months)	Visit 7 (30 months)	Visit 8 (36 months)	Visit 9 (42 months)	Visit 10 (48 months; End of Intervention phase	60days after treatment end	Extended follow up phase (60, 72 & 84 months)
**Clinical assessments and procedures**	Data Collection from hospital records	x	x	x	x	x	x	x	x	x	x	x	x	x
	CT (thorax/abdomen/pelvis)			x*	x	x	x	x		x		x		
	Body Weight	x		x	x	x	x	x	x	x	x	x		
	Adverse event assessment		x	x	x	x	x	x	x	x	x	x	x	
	Concomitant medications	x	x	x	x	x	x	x	x	x	x	x		
	Compliance monitoring		x	x	x	x	x	x	x	x	x	x		
	Participant Questionnaire Pack	x		x	x	x	x	x	x	x	x	x		
	Pregnancy test	x												
	Blood sample for fatty acid levels	x		x	x									
	Biospecimen collection (optional)	x		x	x	x	x	x	x	x	x	x		
**Trial interventions**	Randomisation	x												
	IMP dispensing	x		x	x	x	x	x	x	x	x			

*In the event that macroscopic tumour remains following liver resection and curative treatment is not possible, a CT scan is carried out as soon as possible to measure the extent of existing disease.

Supplementary Figure 1
