# Supplementary material for: Randomised, placebo-controlled, phase 3 trial of the effect of the omega-3 polyunsaturated fatty acid eicosapentaenoic acid (EPA) on colorectal cancer recurrence and survival after surgery for resectable liver metastases: EPA for Metastasis Trial 2 (EMT2) study protocol
Source: BMJ Open. 2023 Nov 29;13(11):e077427. doi: 10.1136/bmjopen-2023-077427 (PMC10689403; doi:10.1136/bmjopen-2023-077427)
Supplement: Supplementary data [file bmjopen-2023-077427supp001.pdf]

A randomised, placebo-controlled, phase 3 trial of the effect of the omega-3 polyunsaturated fatty acid eicosapentaenoic acid (EPA) on colorectal cancer recurrence and survival after surgery for resectable liver metastases: EPA for Metastasis Trial 2 (EMT2) [ClinicalTrials.gov NCT032428477]

Supplementary Data

		Page
1.	List of sites recruiting to EMT2	2
2.	Substantial Amendments to the EMT2 trial protocol	3
3.	Independent members of the Oversight Committees	5

Hull MA, *et al. BMJ Open* 2023; 13:e077427. doi: 10.1136/bmjopen-2023-077427

List of sites recruiting to EMT2

Site name	Date open for recruitment
**Basingstoke** (Hampshire Hospitals NHS Foundation Trust)	21/3/2018
**Birmingham** (University Hospitals Birmingham NHS Trust)	20/7/2018
**Cambridge** (Cambridge University Hospitals NHS Foundation Trust)	30/10/2019
**Cardiff** (University Hospital of Wales, Cardiff and Vale University Health Board)	22/6//2020
**Leeds** (Leeds Teaching Hospitals NHS Trust)	9/5/2018
**Liverpool** (Liverpool University Hospitals NHS Foundation Trust)	21/3/2019
**London** (King's College Hospital NHS Foundation Trust)	4/8/2020
**London** (Royal Free London NHS Foundation Trust)	14/10/2019
**Newcastle** (Newcastle upon Tyne Hospitals NHS Foundation Trust)	28/8/2019
**Nottingham** (Nottingham University Hospitals NHS Trust)	5/12/2018
**Oxford** (Oxford University Hospitals NHS Foundation Trust)	29/6/2022
**Sheffield** (Sheffield Teaching Hospitals NHS Foundation Trust)	11/9/2018
**Southampton** (University Hospital Southampton NHS Foundation Trust)	16/3/2018

Hull MA, *et al. BMJ Open* 2023; 13:e077427. doi: 10.1136/bmjopen-2023-077427

Substantial amendments to the EMT2 trial protocol

Substantial amendment (SA)	Protocol version (submission date)	Reason for the amendment[1]
01	2.0 (21/4/2017)	Use of a new IMP[2] (IPE [active] and mineral oil [placebo] capsules) after approval for use of the IMP (90% EPA triglyceride) used in the seAFOod trial[3] was not granted by MHRA.
02	v3.0 (3/6/20217)	Clarifications in relation to pharmacovigilance measures requested by MHRA.
05	v4.0 (23/8/2018)	Addition of 'remote' (telephone) follow-up and IMP delivery for participants who are unable to attend the recruiting centre for in-person assessment.
06	v5.0 (26/2/2019)	Change of eligibility criteria to remove the stipulation that IMP treatment begins more than 2 weeks before liver resection surgery.<br>Addition of exclusion criterion if current liver metastatic disease has already been treated partially by liver resection surgery.<br>Amendment to allow optional dispensing of IMP at visit 2.
12	v6.0 (13/1/2021)	Inclusion of the option for EMT2 participants to join a translational study to provide stool, blood, urine and tumour tissue for laboratory studies (EMT2 Biospecimen study)
14	v7.0 (25/5/2021)	Urgent Safety Measure - amendment to exclude participants with known soya or peanut allergy and add this to the exclusion criteria for trial screening prompted by guidance in the SmPC for Vazkepa™.<br><br>Amendment of the protocol and PISICF to 1) describe atrial fibrillation risk associated with high-dose omega-3 fatty acids and 2) increase emphasis on increased risk of bleeding in individuals on concomitant anti-thrombotic agents.
15	v8.0 (2/11/2021)	Change of the Reference Safety Information from an Investigator Brochure to the SmPC for Vazkepa™.
16	v9.0 (9/9/2022)	Amendment of sample size estimate and recruitment rate projection.<br>Addition of 'remote' follow-up of participant health care records once IMP is stopped.

[1]Ony Substantial Amendments to the Protocol are listed. Other Substantial Amendments were for addition of new research sites, amendment of the PISICF and use of a trial-specific website.

Hull MA, *et al. BMJ Open* 2023; 13:e077427. doi: 10.1136/bmjopen-2023-077427

[2]Abbreviations: EPA, eicosapentaenoic acid; IMP, Investigative Medicinal Product; IPE, icosapent ethyl; MHRA, Medicines and Healthcare Regulatory Agency; PISICF, Patient Information Sheet and Informed Consent Form; SmPC, Summary of Product Characteristics

[3]The IMP used in the latter stages of the seAFOod polyp prevention trial was soft-gel capsules containing 90% EPA triglyceride (active) or capric/caprylic acids (placebo) – see *Trials* 2013;**14**:237.

Independent members of the Oversight Committees

Trial Steering Committee

Professor Richard Wilson (Chair), University of Glasgow

Professor Louise Brown, University College, London

Professor Vicky Coyle, Queen's University, Belfast

Professor Nick Maynard, Oxford University Hospitals NHS Trust

Patient & Public Representative (details available on request with consent)

Yorkshire Cancer Research (Observer)

Data Monitoring Committee

Professor Robert Steele (Chair), University of Dundee

Professor John Norrie, University of Edinburgh

Professor Christopher Byrne, University of Southampton

Hull MA, *et al. BMJ Open* 2023; 13:e077427. doi: 10.1136/bmjopen-2023-077427
