# Supplementary material for: Randomised, placebo-controlled, phase 3 trial of the effect of the omega-3 polyunsaturated fatty acid eicosapentaenoic acid (EPA) on colorectal cancer recurrence and survival after surgery for resectable liver metastases: EPA for Metastasis Trial 2 (EMT2) study protocol
Source: BMJ Open. 2023 Nov 29;13(11):e077427. doi: 10.1136/bmjopen-2023-077427 (PMC10689403; doi:10.1136/bmjopen-2023-077427)
Supplement: Supplementary data [file bmjopen-2023-077427supp003.pdf]

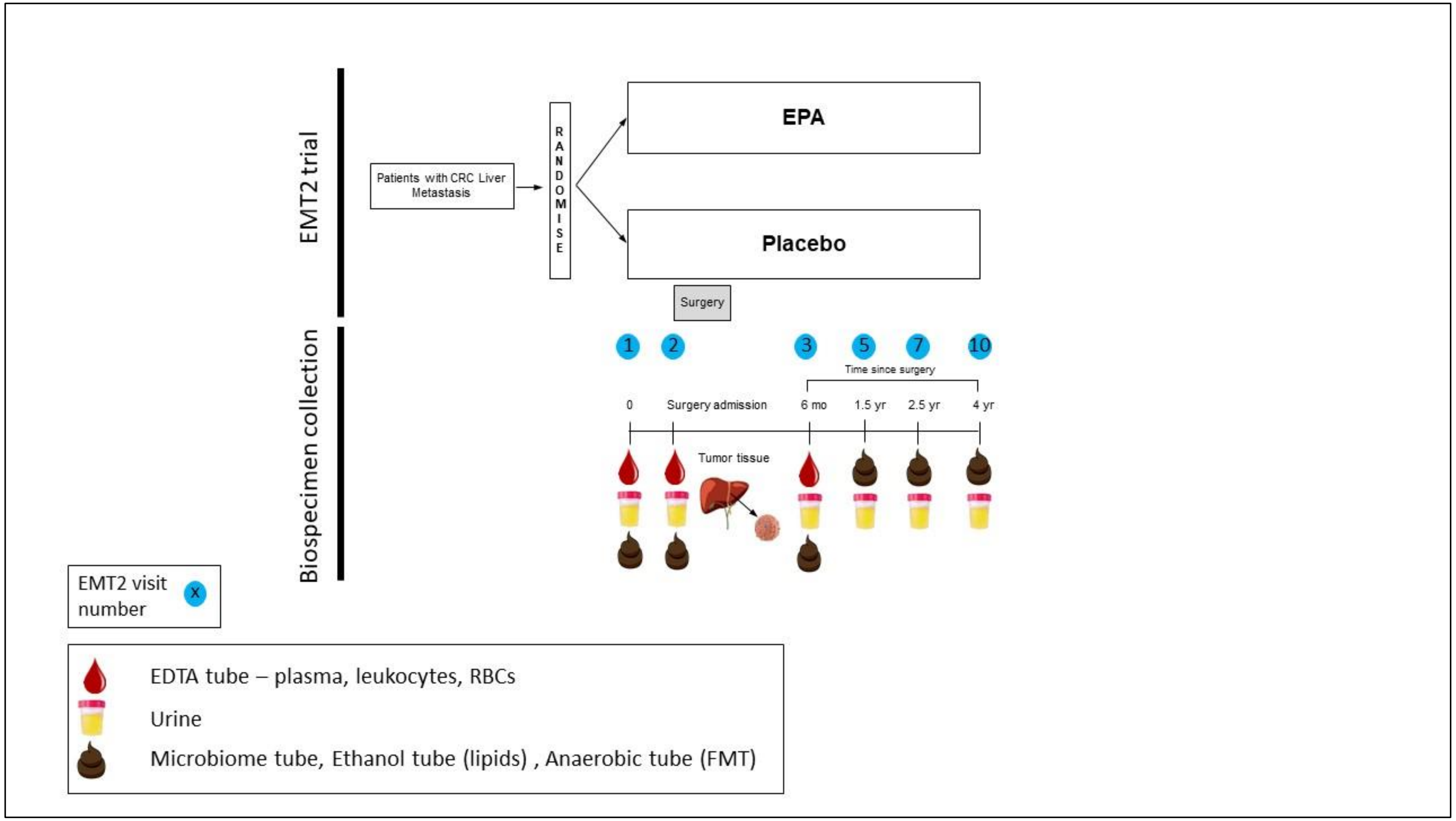

Supplementary Figure 2. Biospecimen collection during EMT2 trial participation. Blood is collected in ethylenediaminetetraacetic acid (EDTA) tubes for immediate fractionation into plasma and red blood cells. A faecal sample is obtained in a DNA Genotek OMNIgeneGUT tube for microbiome analysis, an anaerobic tube for faecal microbial transplant (FMT) studies, and a tube containing 95% ethanol for metabolomic analysis.
