# Supplementary material for: Randomised, placebo-controlled, phase 3 trial of the effect of the omega-3 polyunsaturated fatty acid eicosapentaenoic acid (EPA) on colorectal cancer recurrence and survival after surgery for resectable liver metastases: EPA for Metastasis Trial 2 (EMT2) study protocol
Source: BMJ Open. 2023 Nov 29;13(11):e077427. doi: 10.1136/bmjopen-2023-077427 (PMC10689403; doi:10.1136/bmjopen-2023-077427)
Supplement: Supplementary data [file bmjopen-2023-077427supp004.pdf]

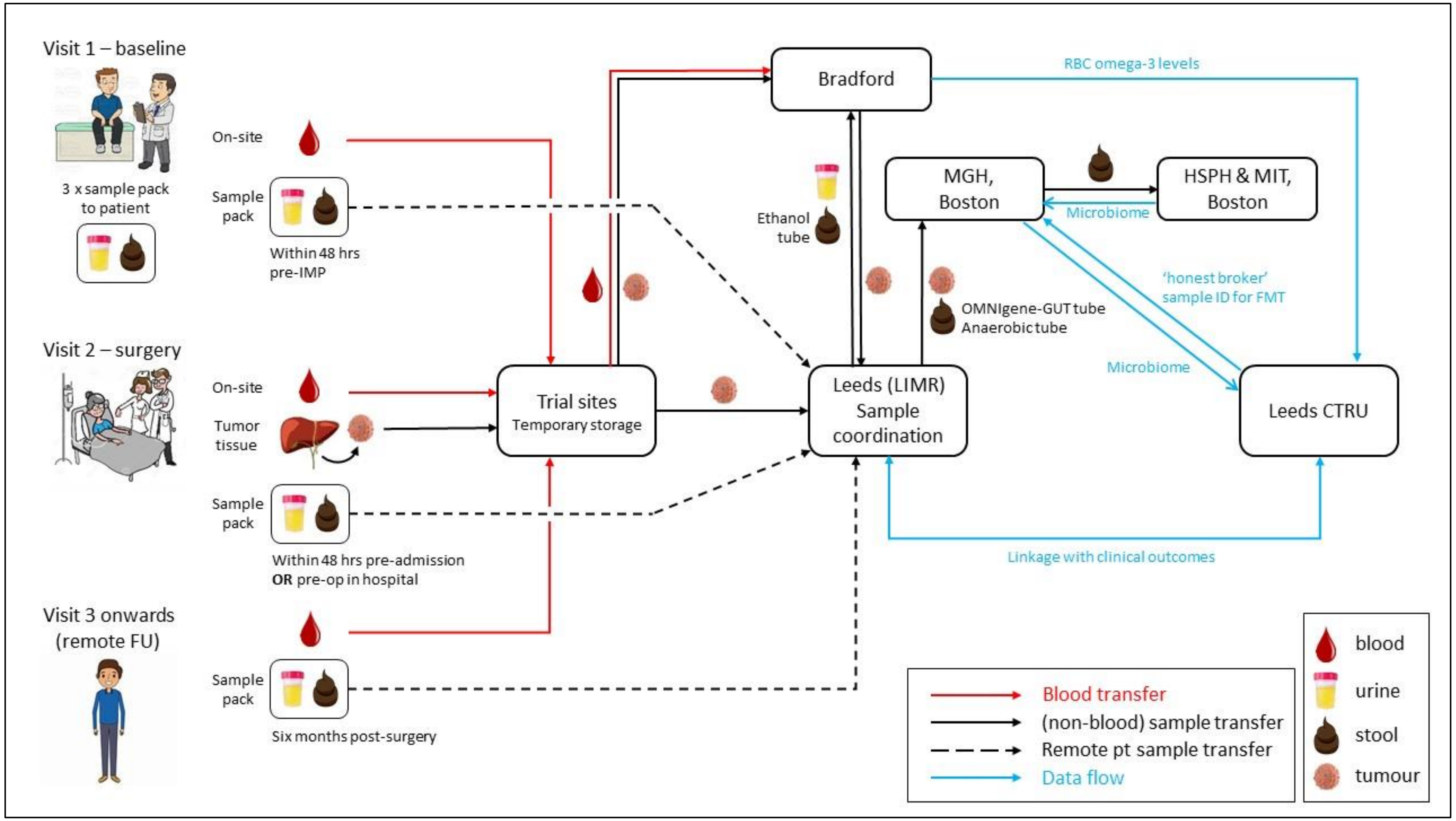

Supplementary Figure 3. Biospecimen and linked data flow during the EMT2 trial. Remote sample transfer from participants to the Leeds Biobank was by Royal Mail Safebox™. Linkage of EMT2 trial outcomes required for design of faecal microbial transplant (FMT) experiments was co-ordinated by the Safety Statistician for EMT2 in order to avoid any disruption of the double-blind for EMT2. CTRU, Clinical Trials Research Unit; HSPH, Harvard School of Public Health; LIMR, Leeds Institute of Medical Research; MGH, Massachussetts General Hospital; MIT, Massachussets Institute of Technology.
