# Supplementary material for: Randomised, placebo-controlled, phase 3 trial of the effect of the omega-3 polyunsaturated fatty acid eicosapentaenoic acid (EPA) on colorectal cancer recurrence and survival after surgery for resectable liver metastases: EPA for Metastasis Trial 2 (EMT2) study protocol
Source: BMJ Open. 2023 Nov 29;13(11):e077427. doi: 10.1136/bmjopen-2023-077427 (PMC10689403; doi:10.1136/bmjopen-2023-077427)
Supplement: Author's manuscript [file bmjopen-2023-077427.draft_revisions.pdf]

BMJ Open

BMJ Open is committed to open peer review. As part of this commitment we make the peer review history of every article we publish publicly available.

When an article is published we post the peer reviewers' comments and the authors' responses online. We also post the versions of the paper that were used during peer review. These are the versions that the peer review comments apply to.

The versions of the paper that follow are the versions that were submitted during the peer review process. They are not the versions of record or the final published versions. They should not be cited or distributed as the published version of this manuscript.

BMJ Open is an open access journal and the full, final, typeset and author-corrected version of record of the manuscript is available on our site with no access controls, subscription charges or pay-per-view fees (http://bmjopen.bmj.com).

If you have any questions on BMJ Open's open peer review process please email info.bmjopen@bmj.com

BMJ Open

A randomised, placebo-controlled, phase 3 trial of the effect of the omega-3 polyunsaturated fatty acid eicosapentaenoic acid (EPA) on colorectal cancer recurrence and survival after surgery for resectable liver metastases: EPA for Metastasis Trial 2 (EMT2)

Journal:	*BMJ Open*
Manuscript ID	bmjopen-2023-077427
Article Type:	Protocol
Date Submitted by the Author:	04-Jul-2023

Complete List of Authors:	Hull, Mark; University of Leeds<br>Ow, Pei Loo; University of Leeds, Leeds Institute of Clinical Trials Research<br>Ruddock, Sharon; University of Leeds, Leeds Institute of Clinical Trials Research<br>Brend, Tim; University of Leeds, Leeds Institute of Medical Research<br>Smith, Alexandra; University of Leeds, Leeds Institute of Clinical Trials Research<br>Marshall, Helen; University of Leeds, Leeds Institute of Clinical Trials Research<br>Song, Mingyang; Harvard University T H Chan School of Public Health<br>Chan, AT; Massachusetts General Hospital<br>Garrett, Wendy; Harvard University T H Chan School of Public Health<br>Yilmaz, Omer; Massachusetts Institute of Technology Koch Institute for Integrative Cancer Research<br>Drew, David; Massachusetts General Hospital<br>Collinson, Fiona; University of Leeds Clinical Trials Research Unit<br>Cockbain, Andrew; Leeds Teaching Hospitals NHS Trust<br>Jones, Robert; Royal Liverpool and Broadgreen University Hospitals NHS Trust<br>Loadman, Paul; University of Bradford, Institute of Cancer Therapeutics<br>Hall, Peter; University of Edinburgh Division of Clinical and Surgical Sciences, Edinburgh Clinical Trials Unit<br>Moriarty, Catherine ; Leeds Teaching Hospitals NHS Trust,<br>Cairns, David; University of Leeds, Leeds Institute of of Clinical Trials Research<br>Toogood, Giles; University of Leeds, Leeds Institute of Medical Research; Leeds Teaching Hospitals NHS Trust

Keywords:	Gastrointestinal tumours < ONCOLOGY, Hepatobiliary surgery < SURGERY, Clinical trials < THERAPEUTICS

For peer review only - http://bmjopen.bmj.com/site/about/guidelines.xhtml

A randomised, placebo-controlled, phase 3 trial of the effect of the omega-3 polyunsaturated fatty acid eicosapentaenoic acid (EPA) on colorectal cancer recurrence and survival after surgery for resectable liver metastases: EPA for Metastasis Trial 2 (EMT2)

Running title: A randomised trial of EPA in patients with colorectal liver metastasis

Mark A Hull[1*]

Pei Loo Ow[2]

Sharon Ruddock[2]

Tim Brend[1]

Alexandra F Smith[2]

Helen Marshall[2]

Mingyang Song[3]

Andrew T Chan[4]

Wendy S Garrett[3]

Omer Yilmaz[5]

David A Drew[4]

Fiona Collinson[2]

Andrew J Cockbain[6]

Robert Jones[7]

Paul M Loadman[8]

Peter S Hall[9]

Catherine Moriarty[6]

David A Cairns[2]

Giles J Toogood[1,6]

[1]Leeds Institute of Medical Research, University of Leeds, Leeds, UK

[2]Leeds Cancer Research UK Clinical Trials Unit, Leeds Institute of Clinical Trials Research, University of Leeds, Leeds, UK

[3]Harvard TH Chan School of Public Health, Boston, USA

[4]Clinical and Translational Epidemiology Unit, Massachusetts General Hospital, Boston, USA

For peer review only

[5]Koch Institute for Integrative Cancer Research at Massachusetts Institute of Technology, Boston, USA

[6]Leeds Teaching Hospitals NHS Trust, Leeds, UK

[7]Royal Liverpool and Broadgreen University Hospitals NHS Trust, Liverpool, UK

[8]Institute for Cancer Therapeutics, University of Bradford, Bradford, UK

[9]Edinburgh Clinical Trials Unit, University of Edinburgh

*Correspondence to Professor Mark Hull, Leeds Institute of Medical Research, Wellcome Trust Brenner Building, St James's University Hospital, Leeds LS9 7TF, United Kingdom (M.A.Hull@leeds.ac.uk)

For peer review only

For peer review only - http://bmjopen.bmj.com/site/about/guidelines.xhtml

Abstract

Introduction: There remains an unmet need for safe and cost-effective adjunctive treatment of advanced colorectal cancer (CRC). The omega-3 polyunsaturated fatty acid eicosapentaenoic acid (EPA) is safe, well-tolerated, and has anti-inflammatory as well as anti-neoplastic properties. A Phase 2 randomised trial of pre-operative EPA free fatty acid 2 g daily in patients undergoing surgery for CRC liver metastasis showed no difference in the primary endpoint (histological tumour proliferation index) compared with placebo. However, the trial demonstrated possible benefit for the pre-specified exploratory endpoint of post-operative disease-free survival. Therefore, we tested the hypothesis that EPA treatment, started before liver resection surgery (and continued post-operatively), improves CRC outcomes in patients with CRC liver metastasis.

Methods and analysis: The EMT2 trial is a randomised, double-blind, placebo-controlled, phase 3 trial of 4g EPA ethyl ester (icosapent ethyl [IPE; Vascepa®]) daily in patients undergoing liver resection surgery for CRC liver metastasis with curative intent. Trial treatment continues for a minimum of two years and maximum of four years, with six-monthly assessments, including Quality of Life outcomes, as well as annual clinical record review after the the trial intervention. The primary endpoint is CRC progression-free survival (PFS). Key secondary endpoints are overall survival, as well as the safety and tolerability of IPE. A minimum 388 participants are estimated to provide 247 CRC progression events during minimum two-year follow-up, allowing detection of a hazard ratio of 0.7 in favour of IPE, with a power of 80% at the 5% (two-sided) level of significance, assuming drop-out of 15%.

Ethics and dissemination: Ethical and Health Research Authority approval was obtained in January 2018. All data will be collected by 2025. Full trial results will be published in 2026. Secondary analyses of health economic data, biomarker studies and other translational work will be published subsequently.

Trial registration number: NCT032428477

For peer review only - http://bmjopen.bmj.com/site/about/guidelines.xhtml

Strengths and limitations of this study

- EMT2 is a randomised, double-blind, placebo-controlled trial of 4 g pure EPA ethyl ester daily
- Treatment starts before CRC liver metastasis surgery and is scheduled for a minimum two years
- The primary endpoint is progression-free survival with a key secondary endpoint of overall survival
- Participant follow-up and IMP delivery can be performed remotely by telephone and courier delivery, which provided COVID-resilience during the Pandemic
- There is a linked biospecimen collection allowing translational studies investigating the mechanism(s) of the anti-CRC activity of EPA

For peer review only - http://bmjopen.bmj.com/site/about/guidelines.xhtml

Introduction

Colorectal cancer (CRC) remains the second most common cause of cancer-related death globally with 935,173 deaths registered in 2020, which represents 9.4% of all cancer deaths worldwide[1]. Only 53% of individuals diagnosed with CRC in England (2013-2017) survive for ten or more years, with the majority of deaths being related to distant metastasis[2]. Approximately half of all CRC patients present with synchronous or metachronous liver metastasis (LM)[3]. Historically, survival of patients with untreated CRCLM was only a few months[4]. Five-year survival rates for CRCLM patients with contemporary multi-modality treatments are now widely of the order of 30%[5]. Surgical resection provides the only prospect of 'cure' for individuals with CRCLM, who are deemed suitable for surgery, with approximately 20% of those undergoing surgery obtaining long-term (>10 years) disease-free survival[6]. However, overall five-year survival following liver resection with curative intent and oxaliplatin-based adjuvant chemotherapy is, at best, 60%[2]. Therefore, there remains an unmet need for safe and cost-effective adjunctive treatment for patients with CRCLM.

The omega-3 polyunsaturated fatty acid (PUFA) eicosapentaenoic acid (EPA) is found naturally in highest quantities in oily fish[7]. It is widely used as a nutritional supplement, most often in combination with the other main marine omega-3 PUFA docosahexaenoic acid (DHA)[7]. Pure EPA ethyl ester is now licensed for use in patients with severe hypertriglyceridaemia and for secondary prophylaxis of vascular events in high-risk individuals taking a statin, who have elevated serum triglyceride levels[8].

Clinical evidence that EPA has anti-CRC activity at early stages of colorectal carcinogenesis is emerging. Treatment with 99% EPA free fatty acid 2 g daily for 6 months was associated with a significant reduction in rectal adenomatous polyp (the benign precursor of CRC) size (29.8%) and multiplicity (22.4%) compared with placebo in a randomised controlled trial (RCT) in patients with familial adenomatous polyposis undergoing sigmoidoscopic surveillance after total colectomy[9]. The seAFOod polyp prevention trial reported that treatment with EPA free fatty acid 2 g daily was associated with a reduction in adenomatous (but not serrated) colorectal polyp recurrence (as measured by colorectal polyp number) in 'high risk' individuals undergoing colonoscopy surveillance in the English Bowel Cancer Screening Programme[10].

Omega-3 PUFAs may also modify the natural history of established CRC. Observational data from cohort studies suggest that increased fish intake is associated with improved survival post-CRC diagnosis, the effect being most prominent for proximal (right-sided) CRCs[11]. The

For peer review only - http://bmjopen.bmj.com/site/about/guidelines.xhtml

EMT study was a phase 2 RCT of EPA free fatty acid 2 g daily in patients awaiting liver resection surgery for CRC liver metastasis[12]. Although the primary endpoint (the histological CRC tumour cell proliferation index) was null, liver metastases in participants from the active EPA arm had a lower vascularity score than placebo-treated tumours, suggesting possible anti-angiogenic activity[12]. In addition, there was an increase in overall survival (OS) and disease-free survival (DFS) in patients randomised to the EPA arm, both of which were specified as exploratory endpoints based on hypotheses that there is prolonged EPA tissue bioavailability due to the slow tissue 'washout' kinetics of EPA and that oral omega-3 PUFA use alters the human gut microbiome[13-14].

There is evidence that concurrent omega-3 PUFA therapy may improve the tolerability and efficacy of cancer chemotherapy from a number of small, heterogeneous studies across several gastrointestinal cancers[15]. Two uncontrolled case series support improved tolerability and nutritional parameters in omega-3 PUFA users during FOLFOX and FOLFIRI regimens for CRC[16-17]. Moreover, a study of omega-3 PUFA-containing oral nutritional supplement (ONS) use demonstrated survival benefit in patients with advanced gastrointestinal cancer receiving cancer chemotherapy and a Glasgow score ≥1 compared with individuals who received no ONS[18]. Systematic review and meta-analysis of the effect of omega-3 PUFA-containing ONS intake on cancer cachexia has highlighted the paucity of high-quality evidence but demonstrated a signal towards anti-inflammatory activity and body weight maintenance for omega-3 PUFA supplementation[19]. Therefore, a valid hypothesis is that EPA treatment abrogates the CRC-related cachexia syndrome characterised by fatigue, anorexia and reduced skeletal muscle mass (sarcopaenia).

In summary, given the promising preliminary data supporting anti-cancer activity of EPA[20], including possible survival benefit for patients with CRCLM[12], and the excellent safety and tolerability profile of EPA[8], we tested the hypothesis that oral treatment with high-dose (4 g daily) pure EPA ethyl ester, started before surgery and continued long-term after liver resection for CRCLM, decreases CRC recurrence, and/or improves overall survival and quality of life, in patients with advanced CRC.

For peer review only

For peer review only - http://bmjopen.bmj.com/site/about/guidelines.xhtml

Trial Design

The EMT2 (**E**PA for **M**etastasis **T**rial **2**) trial is a randomised, double-blind, placebo-controlled, multi-centre, parallel-group, superiority phase 3 trial of EPA in patients undergoing liver resection surgery for CRCLM with curative intent. The trial is based in tertiary-referral Hepatobiliary Surgery Units in England and Wales. Patients are randomised, on an equal basis, to receive either four soft-gel capsules per day each containing 1 g icosapent ethyl (IPE; Vascepa® [USA] or Vazkepa® [UK and Europe], Amarin Pharma Inc., Bridgewater, NJ, USA), taken as two capsules twice daily with food, or four identical placebo capsules (containing light mineral oil[21]) taken in an identical fashion, before liver surgery (Figure 1). The IMP is kindly provided by Amarin Pharma, Inc. and is identical to that used in the REDUCE-IT trial[8] with a daily dose of EPA equivalent to 4 g of EPA-ethyl ester or 3656 mg of EPA free fatty acid). This daily dose of IPE is licensed for use in the USA, UK and Europe for cardiovascular event risk reduction in adult statin-treated patients at high cardiovascular risk with elevated triglycerides (≥ 150 mg/dl [≥ 1.7 mmol/l]) and either established cardiovascular disease, or diabetes and at least one other cardiovascular risk factor.

All participants are scheduled to take Investigational Medicinal Product (IMP) for a minimum of two years and a maximum of four years after liver resection, with continuing annual clinical record review after the end of the intervention phase (Figure 2).

Inclusion and exclusion criteria are listed in Table 1. Patients with synchronous primary and liver metastatic CRC are eligible with recruitment possible at the time of the clinical decision to perform CRCLM surgery. Administration of neo-adjuvant or adjuvant chemotherapy is not an exclusion criterion. Exclusion criteria are also consistent with excipients in the IMP capsule. Consistent with the Summary of Product Characteristics (SmPC) for IPE[22], concurrent use of anti-coagulant or anti-platelet medication is allowed.

The eligibility of individuals, who are participating in another trial, is determined on a case-by-case basis by the research site team, Clinical Trials Unit (CTU) and Sponsor. Co-enrolment with participants in the CRC cohort of the Add-Aspirin trial (ISRCTN74358648) is permitted providing that the respective eligibility criteria of each trial are fulfilled[23].

The current protocol is version 9.0 dated 27th July 2022. The trial website is https://ctru.leeds.ac.uk/EMT2/.

Trial endpoints

For peer review only - http://bmjopen.bmj.com/site/about/guidelines.xhtml

Primary endpoint

The primary endpoint is progression-free survival (PFS) during a minimum of 2 years follow up. Progression-free survival is defined as 1) the time from randomisation to death (from any cause), 2) the first documented evidence of CRC progression (defined as the date of the CT scan or the relevant assessment, at which disease progression or new recurrence is identified and which can be clinical progression or radiological progression evaluated by RECIST1.1[24]), or 3) new CRC recurrence or clinical deterioration unequivocally due to CRC progression. Participants without a CRC progression event will be censored at the time of the last assessment when they were alive and progression-free.

Secondary and exploratory endpoints

The key secondary endpoint is overall survival (OS), which is defined as the time from randomisation to death from any cause. Participants alive at analysis will be censored at the time of the last assessment when they were alive.

Other secondary endpoints are 1) the safety and tolerability of IPE 4 g daily before and after surgery, including during cancer chemotherapy; 2) patient-reported quality of life measures; 3) cost-effectiveness of IPE assessed using a modified UK Cancer Costs Questionnaire Version 2.0; and 4) new primary cancer (excluding non-melanoma skin cancer, breast ductal cancer *in situ*, cervical carcinoma *in situ*, and superficial bladder carcinoma).

An exploratory endpoint is the red blood cell (RBC) membrane EPA level measured at baseline (visit 1), pre-surgery (visit 2) and 6 months after CRCLM surgery (visit 3) from blood samples taken from two Yorkshire research sites (Leeds and Sheffield)[10]. In addition, the skeletal muscle area at the L3 level on CT imaging at 6 months after CRCLM may be measured as another exploratory endpoint dependent on the availability of additional, specific funding for that analysis.

Trial schedule

Potential participants are identified by the Hepatobiliary Multi-disciplinary team (MDT) at the time of the decision to offer CRCLM surgery with curative intent (Figure 1). Potential participants who require a primary CRC resection or 'downsizing' neo-adjuvant chemotherapy for CRCLM prior to surgery are reviewed by the MDT again, following primary management, in order to confirmation up-to-date eligibility. A woman of childbearing potential (WOCBP) is required to undergo a pregnancy test prior to randomisation.

For peer review only - http://bmjopen.bmj.com/site/about/guidelines.xhtml

After written informed consent is obtained, participants are allocated 1:1, using minimisation with a random element, to active or placebo treatment up to the day before CRCLM surgery using an automated 24-hour telephone/online randomisation service based in Leeds CTU. Minimisation is for 1) neo-adjuvant chemotherapy prior to liver resection; 2) known extrahepatic CRC metastases with curative treatment planned; 3) current regular aspirin use or Add-aspirin trial participant still taking IMP; 4) research site; and 5) time between randomisation and date of planned liver resection surgery either <2 weeks or ≥2 weeks.

The schedule of trial assessments before CRCLM surgery, in the peri-operative period, and post-operatively is described in Figure 2 and Supplementary Figure 1. Assessments are carried out every two weeks between randomisation and CRCLM surgery by telephone and on the first day of the hospital stay for CRCLM surgery. Post-operative assessments are six-monthly with a minimum CT Thorax/Abdomen/Pelvis every 6 months for two years followed by annual CT imaging thereafter, in line with routine clinical practice at most sites. Quality of life (QoL) is assessed using EQ-5D-5L, EORTC QLQ-C30 and QLQ-LMC21 supplementary module questionnaires. Health economic outcomes are assessed using a modified UK Cancer Costs Questionnaire Version 2.0 at each trial visit and telephone call.

Participants are followed up for 60 days beyond the end of IMP treatment, followed by annual case record review in order to collect CRC progression and OS data.

Sample size

Prior to the trial opening, 448 participants (224 per arm) were anticipated to give 247 CRC progression events that are required to detect a hazard ratio (HR) of 0.7 in favour of the treatment arm with a power of 80% at the 5% (two-sided) level of significance. The sample size estimate assumed that the control arm would have a median PFS of 21 months and the treatment arm would have a median PFS of 30 months[25-26], with recruitment in a two-year period, minimum two-year follow-up per participant and with 10% drop-out.

The HR for PFS in the Phase II EMT study was 0.694[12], but the EPA arm, by chance, recruited a higher proportion of patients with poor prognostic features, and a multivariate analysis including these factors actually yielded a HR of 0.35 (95% confidence interval: 0.15-0.79) lending credence to the assumed more conservative HR of 0.7 chosen for this larger phase 3

For peer review only - http://bmjopen.bmj.com/site/about/guidelines.xhtml

study, given that the magnitude of benefit observed in phase 2 studies is often greater than that observed in the phase III counterpart.

The EMT study tested EPA-FFA 2g daily[12]. Although the EMT2 study is evaluating IPE 4 g daily, we have assumed at least similar pharmacokinetics of EPA given as IPE, compared with lower-dose EPA-FFA.

Slower than expected recruitment and a higher withdrawal rate due to the COVID-19 pandemic and other factors (see *Trial Progress*) prompted re-evaluation of the sample size required to generate 247 CRC progression events during an extended recruitment period (5.5 years), which provides longer cumulative follow-up. We used a generalized F distribution, rather than exponential function, which more accurately reflected blinded, live PFS data. A simulation study (1000 simulations with random selection of 90% of participants to account for drop-out) in October 2021 predicted that a minimum 388 participants would be required to observe 247 CRC progression events (accounting for actual participant withdrawals, including those related to a pause of the trial at one research site during the first COVID-19 lockdown).

Treatment

Participants receive either four 1 g capsules of IPE or four identical placebo capsules daily (two capsules twice daily with food) from the date of randomisation until the participant has completed study treatment. Treatment is scheduled to continue after disease progression or diagnosis of a new primary cancer. Treatment compliance is assessed at each trial visit or telephone call to determine if the participant has delayed, missed or modified dosing. Unused, expired capsules are collected from participants at a trial visit or at the end of the scheduled intervention period, for counting.

A participant is allowed to take one or more treatment breaks during the trial follow-up, if the participant experiences a persistent adverse reaction (AR), or difficulty taking IMP (for example, because of restrictions to oral intake, during CRC chemotherapy, or due to other concurrent treatment). In the event of a persistent AR, dose reduction (to two capsules daily) is recommended prior to a complete trial treatment break.

'Over-the-counter' nutritional supplements containing any omega-3 PUFA or prescribed omega-3 PUFAs (eg. Omacor®) are prohibited while participants are receiving trial treatment and are an exclusion criterion for the EMT2 trial. Any participant consistently using another

For peer review only - http://bmjopen.bmj.com/site/about/guidelines.xhtml

omega-3 PUFA preparation is withdrawn from trial treatment. Concomitant use of aspirin, other anti-platelet agents, or anticoagulants such as warfarin or a direct-acting oral anticoagulant (DOAC) are allowed, in keeping with the SmPC for IPE (Vazkepa®)[22].

Safety

Reference Safety Information with which to assess seriousness, expectedness and causality for adverse events (AEs) was an Investigator Brochure for IPE (Vascepa®) version 2 dated 1/6/2022 until January 2022, after which the SmPC for IPE (Vazkepa®)[22] was used after the MHRA granted a licence for use of IPE for secondary prophylaxis of vascular events in high-risk individuals taking a statin, who have elevated serum triglyceride levels.

Statistical analysis

A full statistical analysis plan will be written before any analysis is undertaken. Analysis will be conducted on an intention-to-treat basis, in which participants will be included according to the treatment they were randomised to receive, apart from safety/tolerability endpoints which will be based on a safety population of all patients who took at least one dose of any trial treatment.

All hypothesis tests will be two-sided and will use a 5% significance level. No interim futility or early stopping analyses are planned. The final analysis will only take place after the last patient recruited has been followed up for a minimum 24 months, has withdrawn, or died. The primary endpoint (PFS) and the key secondary endpoint (OS) will reported using the 95% confidence interval of the HR. PFS and OS curves will be calculated using the Kaplan-Meier method. Differences in PFS and OS between the treatment groups will be compared using multivariate modelling to adjust for the minimisation factors. Treatment HRs and corresponding 95% confidence intervals will be obtained from the multivariate models.

A subgroup analysis comparing the outcomes for patients that receive ≥2 weeks of IMP prior to surgery and patients that receive <2 weeks of EPA will be performed. Sensitivity analyses for PFS and OS will be conducted to examine the effects of other potential modifiers, in addition to the minimisation factors. Other sensitivity analyses may be carried out for each endpoint to take into account differing assumptions about missing data, if there is a significant

For peer review only - http://bmjopen.bmj.com/site/about/guidelines.xhtml

For peer review only

amount of missing data. Further subgroup and sensitivity analyses may also be performed and will be detailed in the full statistical analysis plan.

Safety information will be summarised by treatment received. The suspected relationship to IPE will be presented along with other causality, the outcome and the event duration. The number and timing of new primary cancers will also be summarised descriptively by treatment group.

QoL measures will be analysed using random effects (multi-level) models to account for the hierarchical nature of repeated measures data and the models will include adjustments for baseline QoL and the minimisation factors. QoL will be presented using mean scores along with the 95% confidence interval. Similar summaries will be produced for quality-adjusted life years (QALYs), as scored by the EQ-5D-5L questionnaire. Cost-effectiveness comparisons between treatment arms will be analysed using the incremental cost effectiveness ratio. The cost per disease recurrence prevented at 2 years and the cost per QALY at 2 years will be reported.

The relationship between the RBC membrane EPA level and both PFS and OS will be analysed using an extended Cox model, which will adjust for the minimisation factors and will also include the RBC membrane EPA level (% of total fatty acids)[10] as a time-dependent covariate.

Trial progress

Health Research Authority approval (Newcastle and North Tyneside REC 16/NE/0140 and MHRA CTA 16767/0289/) was granted on 3/4/2017. EMT2 was prospectively registered with ClinicalTrials.gov (NCT032428477). The European Clinical Trials Database registration is EudraCT 2016-000628-24.

The EMT2 trial opened to recruitment in March 2018. First Participant First Visit was 2/5/2018. The original recruitment period was estimated to be 24 months based on a recruitment projection of 18 patients per month by 8 research sites. This recruitment estimate was based on the annual CRCLM surgery volume at the research sites in 2014 (890 procedures) and a 25% randomisation rate of screened patients. However, the mean monthly recruitment in the first twelve months was four per month. In the early phase of recruitment, 38% of potential participants undergoing screening were ineligible because CRCLM surgery was scheduled within two weeks of randomisation. This prompted a substantial protocol

For peer review only - http://bmjopen.bmj.com/site/about/guidelines.xhtml

amendment to allow recruitment of patients without stipulation about the length of IMP treatment before surgery (Supplementary Data).

The number of research sites has since been expanded to 13 (See Supplementary Data for the list of research sites and their date of opening for recruitment).

The EMT2 trial did not recruit any participants for approximately two months at the onset of the COVID-19 pandemic restrictions in the UK in early 2020, although, in contrast with many randomised trials in the UK, the trial remained open, except for one NHS Trust research site that paused all non-COVID-19 research activity for several months from March 2020. During the COVID-19 pandemic, recruitment continued with wide variations in monthly recruitment rates related to research staff availability (affected by secondment to COVID-19 research roles and sickness), alterations to routine Hepatobiliary Surgery activity and practice, and patient acceptability. The ability to deliver IMP by post and perform trial follow-up visits by telephone aided trial acceptability and retention during the pandemic. We did not observe an increase in trial withdrawal during national COVID-19 lockdowns between March 2020 and mid-2022.

Based on a revised recruitment projection of 5 participants per month, in order to reach a revised recruitment target of 388, the recruitment period has been extended to 67 months (end of recruitment November 2023) with a minimum two-year follow-up. In order to collect the maximum number of CRC progression events from randomised individuals, the protocol was amended to obtain consent from participants at the end of their four-year intervention period to collect data on any CRC progression event from their healthcare record on an annual basis until the end of the follow-up period of the EMT2 trial at the end of November 2025.

Other Protocol amendments during EMT2 trial conduct are listed in Supplementary Data. Key changes during the trial were removal of the stipulation that IMP was started more than two weeks before CRCLM surgery (prompted by realisation that the majority of surgeries were performed within two weeks of the decision to offer CRCLM resection; SA06), and addition of new exclusion criteria related to peanut/soya allergy and sorbitol/xylitol intolerance (SA14).

EMT2 Biospecimen collection and ancillary translational studies

Separate funding was obtained to collect faeces, urine, blood and tumour tissue from EMT2 participants after randomisation to the EMT2 trial for translational laboratory studies. This work is performed under separate HRA approval (REC 20/YH/0306) and ClinicalTrials.gov

For peer review only - http://bmjopen.bmj.com/site/about/guidelines.xhtml

registration (NCT04682665) as the EMT2 Biospecimen collection. The current protocol for Biospecimen collection is version 3.0 dated 28th April 2022.

The EMT2 biospecimen collection is part of a collaboration between the University of Leeds and researchers across several institutions in Boston, USA (Massachussetts General Hospital [MGH], Harvard TH Chan School of Public Health [HSPH] and Massachussetts Institute of Technology [MIT]) and the University of Bradford, funded by National Institutes of Health to investigate the role of the gut microbiota and its relationship with the host anti-tumour immune response in the anti-CRC activity of EPA.

Consent to provide biospecimens is obtained from new EMT2 participants, only after EMT2 entry. Faecal, blood and urine samples are collected 1) after EMT2 trial randomisation, before starting active EPA or placebo, 2) just before surgery, and 3) at 6-months after CRCLM surgery (Supplementary Figure 2), with the option to provide further specimens during EMT2 follow-up out to four years. It is also possible for existing EMT2 participants to provide a 'one-off' faecal and urine sample at any time whilst taking IMP during the follow-up period. There are five participating sites, from which baseline (visit 1) and surgery (visit 2) samples are transferred to the Biobank in Leeds. We use a modified Micro-N collection tool[27], which facilitates collection of faecal specimens in a DNA Genotek Inc. OMNIgene.GUT tube (for metagenome and metatranscriptome profiling), in 95% ethanol (for faecal metabolomic analysis), and an anaerobic tube filled anaerobically with liquid dental transport medium (for culture and gnotobiotic animal model studies), for postal delivery to the Leeds Biobank, alongside a urine sample (Supplementary Figure 2).

Laboratory studies will take place in Boston, Leeds and Bradford (Supplementary Figure 3). Linked, fully anonymised data (including EMT2 survival outcomes) will be shared between Leeds and MGH using the EMT2 trial Safety Statistician as the 'honest broker' in order to avoid inadvertent disclosure of individual treatment allocation to EMT2 Investigators (Supplementary Figure 3).

Patient and Public Involvement

A Patient representative was involved in trial design and is a member of the Trial Management Group. This Patient representative has supported design of all patient-facing materials including website content and Instructions for Biospecimen collection. A different Patient representative sits on the Trial Steering Committee.

For peer review only - http://bmjopen.bmj.com/site/about/guidelines.xhtml

References

1. Ferlay J, Ervik M, Lam F, Colombet M, Mery L, Piñeros M, Znaor A, Soerjomataram I, Bray F (2020). Global Cancer Observatory: Cancer Today. Lyon, France: International Agency for Research on Cancer. Available from: https://gco.iarc.fr/today, accessed [21 June 2023].
2. Cancer Research UK CancerStats. http://www.cancerresearchuk.org/cancer-info/cancerstats/types/bowel/ (accessed 21 June 2023).
3. Hackl C, Neumann P, Gerken M, Loss M, Klinkhammer-Schalke M, Schlitt HJ. Treatment of colorectal liver metastases in Germany: a ten-year population-based analysis of 5772 cases of primary colorectal adenocarcinoma. BMC Cancer 2014;14:810
4. Bengtsson G, Carlsson G, Hafström L, Jönsson PE. Natural history of patients with untreated liver metastases from colorectal cancer. Am J Surg 1981;141:586-589.
5. Roberts KJ, White A, Cockbain A, *et al*. Performance of prognostic scores in predicting long-term outcome following resection of colorectal liver metastases. Br J Surg 2014;101:856-866.
6. Creasy JM, Sadot E, Koerkamp BG, Chou JF, Gonen M, Kemeny NE, Balachandran VP, Kingham TP, DeMatteo RP, Allen PJ, Blumgart LH, Jarnagin WR, D'Angelica MI. Actual 10-year survival after hepatic resection of colorectal liver metastases: what factors preclude cure? Surgery. 2018;163:1238-1244.
7. Cholewski M, Tomczykowa M, Tomczyk M. A Comprehensive Review of Chemistry, Sources and Bioavailability of Omega-3 Fatty Acids. Nutrients 2018;10:1662.
8. Bhatt DL, Steg PG, Miller M, *et al*. Cardiovascular risk reduction with icosapent ethyl for hypertriglceridemia. N Engl J Med 2019;380:11-22.
9. West NJ, Clark SK, Phillips RKS, *et al*. Eicosapentaenoic acid reduces rectal polyp number and size in familial adenomatous polyposis. Gut 2010;59:918-925.
10. Hull MA, Sprange K, Hepburn T, *et al*. Eicosapentaenoic acid and aspirin, alone and in combination, for the prevention of colorectal adenomas (seAFOod Polyp Prevention trial): a multicentre, randomised, double-blind, placebo-controlled, 2 × 2 factorial trial. Lancet 2018;392:2583-2594.
11. Song M, Chan AT. Environmental Factors, Gut Microbiota, and Colorectal Cancer Prevention. Clin Gastroenterol Hepatol 2019;17:275-289.

For peer review only - http://bmjopen.bmj.com/site/about/guidelines.xhtml

12. Cockbain AJ, Volpato M, Race AD, *et al*. Anti-colorectal cancer activity of the omega-3 polyunsaturated fatty acid eicosapentaenoic acid. Gut 2014;63:1760-1768.
13. Cao J, Schwichtenberg KA, Hanson NQ, *et al*. Incorporation and clearance of omega-3 fatty acids in erythrocyte membranes and plasma phospholipids. Clin Chem 2006;52:2265-2272.
14. Watson H, Mitra S, Croden FC, *et al*. A randomised trial of the effect of omega-3 polyunsaturated fatty acid supplements on the human intestinal microbiota. *Gut* 2018;67:1974-1983.
15. de van der Schueren MAE, Laviano A, Blanchard H, *et al*. Systematic review and meta-analysis of the evidence for oral nutritional intervention on nutritional and clinical outcomes during chemo(radio)therapy: current evidence and guidance for design of future trials. Ann Oncol 2018;29:1141-1153.
16. Trabal J, Leyes P, Forga M, *et al*. Potential usefulness of an EPA-enriched nutritional supplement on chemotherapy tolerability in cancer patients without overt malnutrition. Nutr Hosp 2010;25:736-740.
17. Read JA, Beale PJ, Volker DH, *et al*. Nutrition intervention using an eicosapentaenoic acid (EPA)-containing supplement in patients with advanced colorectal cancer. Effects on nutritional and inflammatory status: a phase II trial. Support Care Cancer 2007;15:301-307.
18. Shirai Y, Okugawa Y, Hishida A, *et al*. Fish oil-enriched nutrition combined with systemic chemotherapy for gastrointestinal cancer patients with cancer cachexia. Sci Rep 2017;7:4826.
19. de Castro GS, Andrade MF, Pinto FCS, *et al*. Omega-3 fatty acid supplementation and its impact on systemic inflammation and body weight in patients with cancer cachexia – A systematic review and meta-analysis. Front Nutr 2022;8:797513.
20. Aldoori J, Cockbain AJ, Toogood GJ, *et al*. Omega-3 polyunsaturated fatty acids: moving towards precision use for prevention and treatment of colorectal cancer. Gut 2022;71:822-837.
21. Olshansky B, Chung MK, Budoff MJ, *et al*. Mineral oil: safety and use as placebo in REDUCE-IT and other clinical studies. Eur Heart J Suppl 2020;22:J34–J48.
22. Summary of Product Characteristics for Icosapent Ethyl (Vazkepa®). https://www.medicines.org.uk/emc/product/12964

For peer review only - http://bmjopen.bmj.com/site/about/guidelines.xhtml

23. Coyle C, Cafferty FH, Rowley S, *et al*. ADD-ASPIRIN: A phase III, double-blind, placebo controlled, randomised trial assessing the effects of aspirin on disease recurrence and survival after primary therapy in common non-metastatic solid tumours. Contemp Clin Trials 2016;51:56-64.
24. Eisenhauer EA, Therasse P, Bogaerts J, *et al.* New response criteria in solid tumours: revised RECIST guideline (version 1.1). Eur J Cancer 2009;45:228-247.
25. Nordlinger B, Sorbye H, Glimelius B, *et al*. Perioperative chemotherapy with FOLFOX4 and surgery versus surgery alone for resectable liver metastases from colorectal cancer (EORTC Intergroup trial 40983): a randomised controlled trial. Lancet 2008;371:1007-1016.
26. Primrose J, Falk S, Finch-Jones M, *et al*. Systemic chemotherapy with or without cetuximab in patients with resectable colorectal liver metastasis: the New EPOC randomised controlled trial. Lancet Oncol 2014;15:601-611.
27. Everett C, Li C, Wilkinson JE, *et al.* Overview of the Microbiome Among Nurses study (Micro-N) as an example of prospective characterization of the microbiome within cohort studies. Nat Protoc 2021;16:2724–2731.

For peer review only

For peer review only - http://bmjopen.bmj.com/site/about/guidelines.xhtml

Acknowledgements

The Trial Management Group wish to thank Amarin Pharma, Inc. for providing active and placebo IMP for the EMT2 trial. Amarin Pharma, Inc. played no role in the design or conduct of the EMT2 trial. The support of the Clinical Trials Research Unit (CTRU) at the University of Leeds is essential to the successful running of the study; we thank all CTRU staff who have contributed, past and present. The Trial Management Group also wish to acknowledge the important role played by the Patient & Public Representatives on the Trial Management Group and Trial Steering Committee.

Authors' contributions

MAH is the Chief Investigator. **MAH:** Conceptualization, Investigation, Resources, Writing – Original Draft, Writing – Review & Editing, Supervision, Project Administration, Funding acquisition **PLO:** Methodology, Formal analysis, Investigation, Data Curation, Writing – Original Draft, Writing – Review & Editing **SR:** Investigation, Writing – Review & Editing, Visualization **TB:** Investigation, Resources, Data Curation, Writing – Review & Editing **AFS:** Methodology Investigation, Data Curation, Writing – Review & Editing, Supervision, Project Administration **HM:** Methodology, Formal analysis **MS:** Conceptualization, Resources, Writing – Review & Editing, Funding acquisition **ATC:** Conceptualization, Resources, Writing – Review & Editing, Supervision, Project Administration, Funding acquisition **WSG:** Conceptualization, Investigation, Resources, Writing – Review & Editing, Funding acquisition **OY:** Conceptualization, Investigation, Resources, Writing – Review & Editing, Funding acquisition **DAD:** Investigation, Data Curation **FC:** Conceptualization, Writing – Review & Editing, Funding acquisition **AJC:** Investigation, Resources, Writing – Review & Editing **RJ:** Investigation, Resources, Writing – Review & Editing **PML:** Methodology, Investigation, Writing – Review & Editing, Supervision, Funding acquisition **PSH:** Methodology, Resources, Writing – Review & Editing, Funding acquisition **CM:** Investigation, Writing – Review & Editing **DAC:** Methodology, Formal analysis, Investigation, Data Curation, Writing – Review & Editing, Supervision, Project Administration **GJT:** Conceptualization, Investigation, Resources, Writing – Review & Editing, Supervision, Project Administration, Funding acquisition

For peer review only

Funding

The EMT2 trial is funded by Yorkshire Cancer Research (L387) and is sponsored by the University of Leeds. The EMT2 Biospecimen collection is funded by the National Institutes of Health (1R01CA243454-01A1) and is sponsored by the University of Leeds. Both studies have been adopted to the NIHR Clinical Research Network (CRN) Portfolio (CPMS ID 34700 and 47372, respectively) and have benefited from CRN research staff support.

The funder and the Sponsor had no role in the design of the trial, data collection, or input into the writing of this manuscript.

Competing interests

The Authors declare no Conflict of Interest

For peer review only - http://bmjopen.bmj.com/site/about/guidelines.xhtml

Figure Legends

Figure 1. Pathway for screening, obtaining informed consent and randomisation to the EMT2 trial. *the full list of inclusion and exclusion criteria are listed in Table 1. CRC, colorectal cancer; CRCLM, colorectal cancer liver metastasis; IMP, Investigational Medicinal Product; MDT, Multi-disciplinary team meeting; O3FA, omega-3 fatty acid.

Figure 2. Assessment schedule from randomisation onwards in the EMT2 trial. *the EMT2 Biospecimen collection is a distinct study with separate ethical approval and study registration (see text). IMP, Investigational Medicinal Product; IPE, icosapent ethyl.

For peer review only

For peer review only - http://bmjopen.bmj.com/site/about/guidelines.xhtml

Inclusion criteria	Exclusion criteria
Age ≥ 18 years	Previous surgery for the management of current CRCLM†
Able to provide written informed consent	Incurable extra-hepatic metastases
Histological diagnosis of CRC with evidence of one or more liver metastases	Current (in the last two months) or planned regular (more than three doses per week) use of omega-3 PUFA-containing medication or supplements
Planned liver resection surgery for CRCLM with curative intent*	Fish/seafood allergy
Intention to receive IMP treatment prior to CRCLM surgery	hereditary fructose intolerance
	Soya or peanut allergy
	Inability to comply with trial schedule
	Known bleeding tendency (eg. von Willebrand disease)
	A previous malignancy within 5 years‡
	Pregnant/breast-feeding woman or WOCBP not willing to use effective contraceptive measures**
	Fertile (post-pubescent and not permanently sterile by vasectomy or bilateral orchidectomy) man not willing to use barrier contraception

Table 1. Inclusion and exclusion criteria for the EMT2 trial. *includes repeat CRCLM liver surgery (a second independent liver resection) for a separate metachronous CRC liver recurrence. †Patients who have already undergone one CRCLM surgery as part of a two-stage procedure are ineligible. ‡other than CRC, non-melanoma skin cancer where treatment consisted of resection only or radiotherapy, ductal breast carcinoma *in situ* where treatment consisted of resection only, cervical carcinoma *in situ* where treatment consisted of resection

For peer review only - http://bmjopen.bmj.com/site/about/guidelines.xhtml

only, and superficial bladder carcinoma where treatment consisted of resection only. **WOCBP, woman of child-bearing potential: Effective contraception includes combined (oestrogen- and progesterone-containing) hormonal contraception, progesterone-only hormonal contraception, intrauterine device, intrauterine hormone-releasing system, bilateral tubal occlusion, vasectomised partner, practicing true sexual abstinence.

For peer review only

For peer review only - http://bmjopen.bmj.com/site/about/guidelines.xhtml

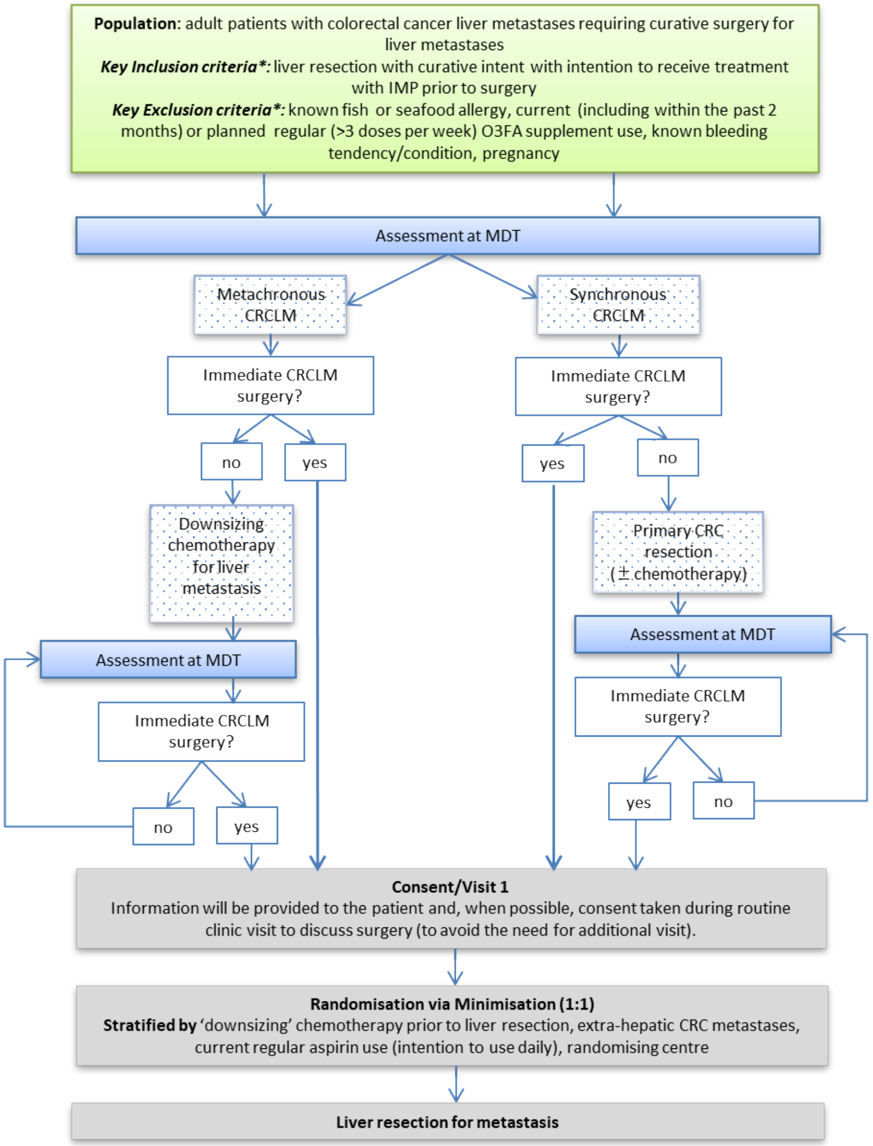
Population: adult patients with colorectal cancer liver metastases requiring curative surgery for liver metastases
Key Inclusion criteria*: liver resection with curative intent with intention to receive treatment with IMP prior to surgery
Key Exclusion criteria*: known fish or seafood allergy, current (including within the past 2 months) or planned regular (>3 doses per week) O3FA supplement use, known bleeding tendency/condition, pregnancy
Assessment at MDT
Metachronous CRCLM
Synchronous CRCLM
Immediate CRCLM surgery?
Immediate CRCLM surgery?
no
yes
yes
no
Downsizing chemotherapy for liver metastasis
Primary CRC resection (± chemotherapy)
Assessment at MDT
Assessment at MDT
Immediate CRCLM surgery?
Immediate CRCLM surgery?
no
yes
yes
no
Consent/Visit 1
Information will be provided to the patient and, when possible, consent taken during routine clinic visit to discuss surgery (to avoid the need for additional visit).
Randomisation via Minimisation (1:1)
Stratified by 'downsizing' chemotherapy prior to liver resection, extra-hepatic CRC metastases, current regular aspirin use (intention to use daily), randomising centre
Liver resection for metastasis

Figure 1

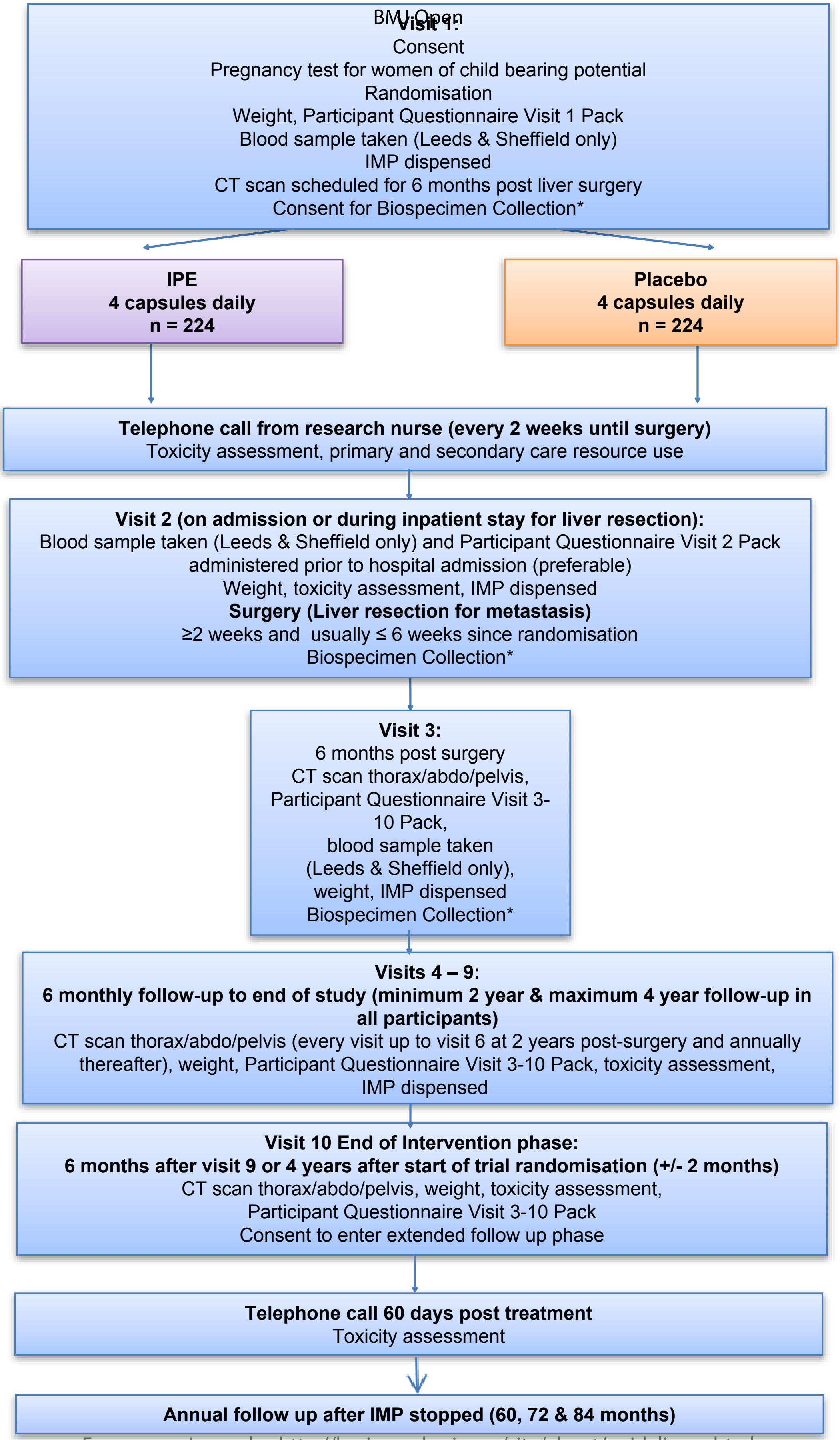

For peer review only - http://bmjopen.bmj.com/site/about/guidelines.xhtml

Figure 2

A randomised, placebo-controlled, phase 3 trial of the effect of the omega-3 polyunsaturated fatty acid eicosapentaenoic acid (EPA) on colorectal cancer recurrence and survival after surgery for resectable liver metastases: EPA for Metastasis Trial 2 (EMT2) [ClinicalTrials.gov NCT032428477]

Supplementary Data

	Page
1. List of sites recruiting to EMT2	2
2. Substantial Amendments to the EMT2 trial protocol	3
3. Independent members of the Oversight Committees	5

For peer review only

List of sites recruiting to EMT2

Site name	Date open for recruitment
**Basingstoke** (Hampshire Hospitals NHS Foundation Trust)	21/3/2018
**Birmingham** (University Hospitals Birmingham NHS Trust)	20/7/2018
**Cambridge** (Cambridge University Hospitals NHS Foundation Trust)	30/10/2019
**Cardiff** (University Hospital of Wales, Cardiff and Vale University Health Board)	22/6//2020
**Leeds** (Leeds Teaching Hospitals NHS Trust)	9/5/2018
**Liverpool** (Liverpool University Hospitals NHS Foundation Trust)	21/3/2019
**London** (King's College Hospital NHS Foundation Trust)	4/8/2020
**London** (Royal Free London NHS Foundation Trust)	14/10/2019
**Newcastle** (Newcastle upon Tyne Hospitals NHS Foundation Trust)	28/8/2019
**Nottingham** (Nottingham University Hospitals NHS Trust)	5/12/2018
**Oxford** (Oxford University Hospitals NHS Foundation Trust)	29/6/2022
**Sheffield** (Sheffield Teaching Hospitals NHS Foundation Trust)	11/9/2018
**Southampton** (University Hospital Southampton NHS Foundation Trust)	16/3/2018

For peer review only

For peer review only - http://bmjopen.bmj.com/site/about/guidelines.xhtml

Substantial amendments to the EMT2 trial protocol

Substantial amendment (SA)	Protocol version (submission date)	Reason for the amendment[1]
01	2.0 (21/4/2017)	Use of a new IMP[2] (IPE [active] and mineral oil [placebo] capsules) after approval for use of the IMP (90% EPA triglyceride) used in the seAFOod trial[3] was not granted by MHRA.
02	v3.0 (3/6/20217)	Clarifications in relation to pharmacovigilance measures requested by MHRA.
05	v4.0 (23/8/2018)	Addition of 'remote' (telephone) follow-up and IMP delivery for participants who are unable to attend the recruiting centre for in-person assessment.
06	v5.0 (26/2/2019)	Change of eligibility criteria to remove the stipulation that IMP treatment begins more than 2 weeks before liver resection surgery.<br>Addition of exclusion criterion if current liver metastatic disease has already been treated partially by liver resection surgery.<br>Amendment to allow optional dispensing of IMP at visit 2.
12	v6.0 (13/1/2021)	Inclusion of the option for EMT2 participants to join a translational study to provide stool, blood, urine and tumour tissue for laboratory studies (EMT2 Biospecimen study)
14	v7.0 (25/5/2021)	Urgent Safety Measure - amendment to exclude participants with known soya or peanut allergy and add this to the exclusion criteria for trial screening prompted by guidance in the SmPC for Vazkepa™.<br><br>Amendment of the protocol and PISICF to 1) describe atrial fibrillation risk associated with high-dose omega-3 fatty acids and 2) increase emphasis on increased risk of bleeding in individuals on concomitant anti-thrombotic agents.
15	v8.0 (2/11/2021)	Change of the Reference Safety Information from an Investigator Brochure to the SmPC for Vazkepa™.
16	v9.0 (9/9/2022)	Amendment of sample size estimate and recruitment rate projection.<br>Addition of 'remote' follow-up of participant health care records once IMP is stopped.

[1]Ony Substantial Amendments to the Protocol are listed. Other Substantial Amendments were for addition of new research sites, amendment of the PISICF and use of a trial-specific website.

For peer review only - http://bmjopen.bmj.com/site/about/guidelines.xhtml

[2]Abbreviations: EPA, eicosapentaenoic acid; IMP, Investigative Medicinal Product; IPE, icosapent ethyl; MHRA, Medicines and Healthcare Regulatory Agency; PISICF, Patient Information Sheet and Informed Consent Form; SmPC, Summary of Product Characteristics

[3]The IMP used in the latter stages of the seAFOod polyp prevention trial was soft-gel capsules containing 90% EPA triglyceride (active) or capric/caprylic acids (placebo) – see *Trials* 2013;**14**:237.

For peer review only

Independent members of the Oversight Committees

Trial Steering Committee

Professor Richard Wilson (Chair), University of Glasgow

Professor Louise Brown, University College, London

Professor Vicky Coyle, Queen's University, Belfast

Professor Nick Maynard, Oxford University Hospitals NHS Trust

Patient & Public Representative (details available on request with consent)

Yorkshire Cancer Research (Observer)

Data Monitoring Committee

Professor Robert Steele (Chair), University of Dundee

Professor John Norrie, University of Edinburgh

Professor Christopher Byrne, University of Southampton

For peer review only

		Visit 1 (randomisation)	Fortnightly pre-op telephone call	Visit 2: Surgery	Visit 3 (6 months)*	Visit 4 (12 months)	Visit 5 (18 months)	Visit 6 (24 months)	Visit 7 (30 months)	Visit 8 (36 months)	Visit 9 (42 months)	Visit 10 (48 months; End of Intervention phase	60days after treatment end	Extended follow up phase (60, 72 & 84 months)
**Clinical assessments and procedures**	Data Collection from hospital records	x	x	x	x	x	x	x	x	x	x	x	x	x
	CT (thorax/abdomen/pelvis)			x*	x	x	x	x		x		x		
	Body Weight	x		x	x	x	x	x	x	x	x	x		
	Adverse event assessment		x	x	x	x	x	x	x	x	x	x	x	
	Concomitant medications	x	x	x	x	x	x	x	x	x	x	x		
	Compliance monitoring		x	x	x	x	x	x	x	x	x	x		
	Participant Questionnaire Pack	x		x	x	x	x	x	x	x	x	x		
	Pregnancy test	x												
	Blood sample for fatty acid levels	x		x	x									
	Biospecimen collection (optional)	x		x	x	x	x	x	x	x	x	x		
**Trial interventions**	Randomisation	x												
	IMP dispensing	x		x	x	x	x	x	x	x	x			

*In the event that macroscopic tumour remains following liver resection and curative treatment is not possible, a CT scan is carried out as soon as possible to measure the extent of existing disease.

Supplementary Figure 1

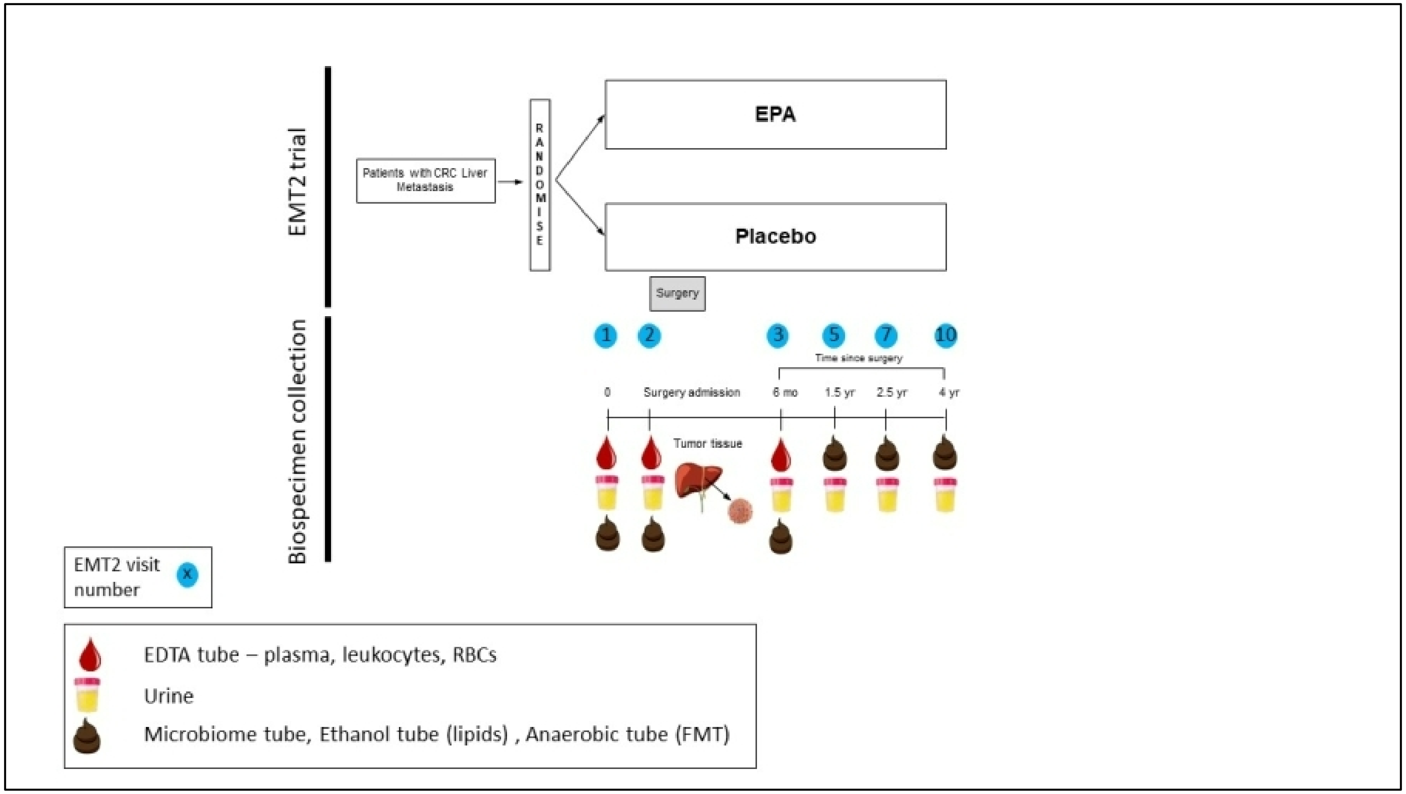

Supplementary Figure 2. Biospecimen collection during EMT2 trial participation. Blood is collected in ethylenediaminetetraacetic acid (EDTA) tubes for immediate fractionation into plasma and red blood cells. A faecal sample is obtained in a DNA Genotek OMNIgeneGUT tube for microbiome analysis, an anaerobic tube for faecal microbial transplant (FMT) studies, and a tube containing 95% ethanol for metabolomic analysis.

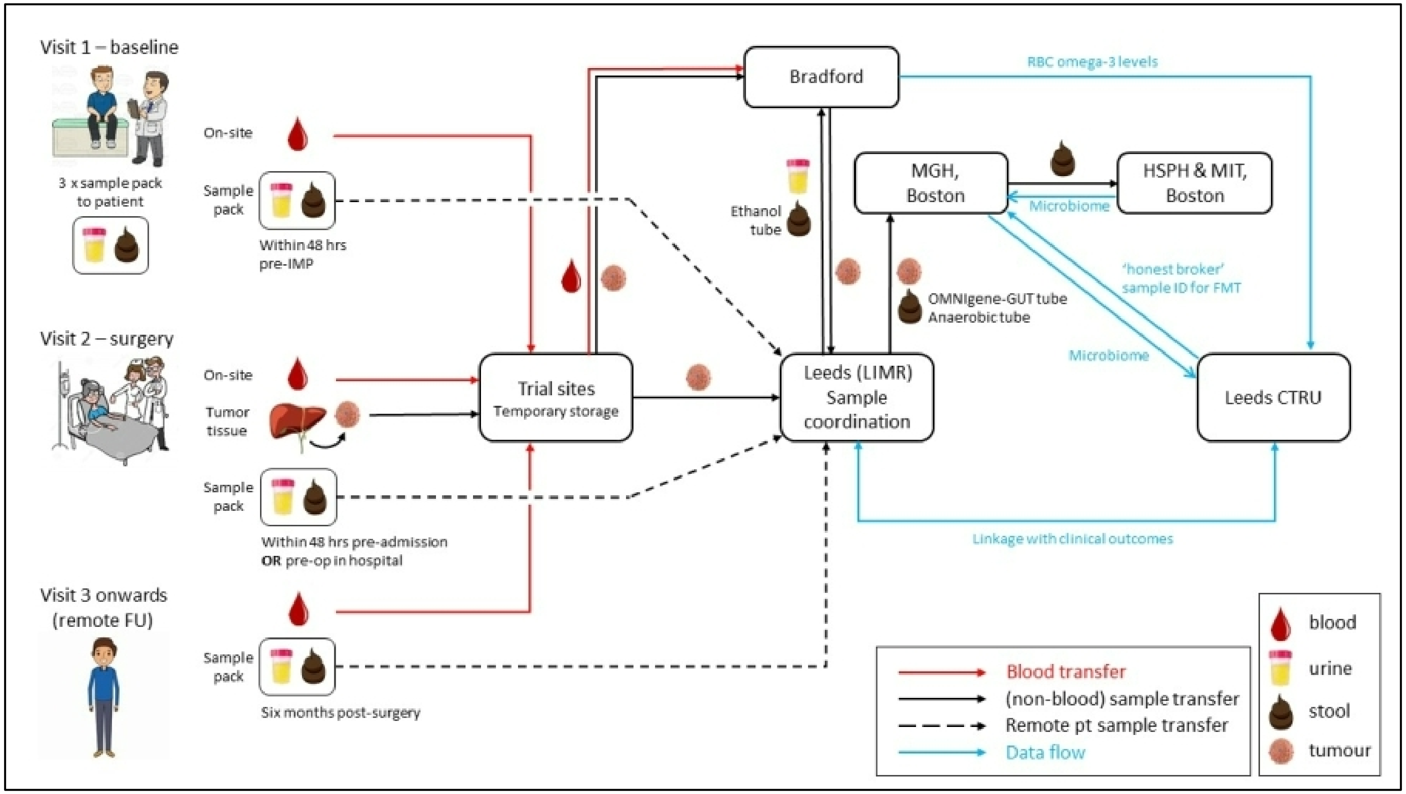

Supplementary Figure 3. Biospecimen and linked data flow during the EMT2 trial. Remote sample transfer from participants to the Leeds Biobank is by Royal Mail Safebox™. Linkage of EMT2 trial outcomes required for design of faecal microbial transplant (FMT) experiments is co-ordinated by the Safety Statistician for EMT2 in order to avoid any disruption of the double-blind for EMT2. CTRU, Clinical Trials Research Unit; HSPH, Harvard School of Public Health; LIMR, Leeds Institute of Medical Research; MGH, Massachussetts General Hospital; MIT, Massachussets Institute of Technology.

SPIRIT

STANDARD PROTOCOL ITEMS: RECOMMENDATIONS FOR INTERVENTIONAL TRIALS

SPIRIT 2013 Checklist: Recommended items to address in a clinical trial protocol and related documents*

Section/item	Item No	Description
**Administrative information**		
Title	1	Descriptive title identifying the study design, population, interventions, and, if applicable, trial acronym
Trial registration	2a	Trial identifier and registry name. If not yet registered, name of intended registry
	2b	All items from the World Health Organization Trial Registration Data Set
Protocol version	3	Date and version identifier
Funding	4	Sources and types of financial, material, and other support
Roles and responsibilities	5a	Names, affiliations, and roles of protocol contributors
	5b	Name and contact information for the trial sponsor
	5c	Role of study sponsor and funders, if any, in study design; collection, management, analysis, and interpretation of data; writing of the report; and the decision to submit the report for publication, including whether they will have ultimate authority over any of these activities
	5d	Composition, roles, and responsibilities of the coordinating centre, steering committee, endpoint adjudication committee, data management team, and other individuals or groups overseeing the trial, if applicable (see Item 21a for data monitoring committee)
**Introduction**		
Background and rationale	6a	Description of research question and justification for undertaking the trial, including summary of relevant studies (published and unpublished) examining benefits and harms for each intervention
	6b	Explanation for choice of comparators
Objectives	7	Specific objectives or hypotheses
Trial design	8	Description of trial design including type of trial (eg, parallel group, crossover, factorial, single group), allocation ratio, and framework (eg, superiority, equivalence, noninferiority, exploratory)

For peer review only

Methods: Participants, interventions, and outcomes

Study setting	9	Description of study settings (eg, community clinic, academic hospital) and list of countries where data will be collected. Reference to where list of study sites can be obtained
Eligibility criteria	10	Inclusion and exclusion criteria for participants. If applicable, eligibility criteria for study centres and individuals who will perform the interventions (eg, surgeons, psychotherapists)
Interventions	11a	Interventions for each group with sufficient detail to allow replication, including how and when they will be administered
	11b	Criteria for discontinuing or modifying allocated interventions for a given trial participant (eg, drug dose change in response to harms, participant request, or improving/worsening disease)
	11c	Strategies to improve adherence to intervention protocols, and any procedures for monitoring adherence (eg, drug tablet return, laboratory tests)
	11d	Relevant concomitant care and interventions that are permitted or prohibited during the trial
Outcomes	12	Primary, secondary, and other outcomes, including the specific measurement variable (eg, systolic blood pressure), analysis metric (eg, change from baseline, final value, time to event), method of aggregation (eg, median, proportion), and time point for each outcome. Explanation of the clinical relevance of chosen efficacy and harm outcomes is strongly recommended
Participant timeline	13	Time schedule of enrolment, interventions (including any run-ins and washouts), assessments, and visits for participants. A schematic diagram is highly recommended (see Figure)
Sample size	14	Estimated number of participants needed to achieve study objectives and how it was determined, including clinical and statistical assumptions supporting any sample size calculations
Recruitment	15	Strategies for achieving adequate participant enrolment to reach target sample size

Methods: Assignment of interventions (for controlled trials)

Allocation:

Sequence generation	16a	Method of generating the allocation sequence (eg, computer-generated random numbers), and list of any factors for stratification. To reduce predictability of a random sequence, details of any planned restriction (eg, blocking) should be provided in a separate document that is unavailable to those who enrol participants or assign interventions

Allocation concealment mechanism	16b	Mechanism of implementing the allocation sequence (eg, central telephone; sequentially numbered, opaque, sealed envelopes), describing any steps to conceal the sequence until interventions are assigned
Implementation	16c	Who will generate the allocation sequence, who will enrol participants, and who will assign participants to interventions
Blinding (masking)	17a	Who will be blinded after assignment to interventions (eg, trial participants, care providers, outcome assessors, data analysts), and how
	17b	If blinded, circumstances under which unblinding is permissible, and procedure for revealing a participant's allocated intervention during the trial
**Methods: Data collection, management, and analysis**		
Data collection methods	18a	Plans for assessment and collection of outcome, baseline, and other trial data, including any related processes to promote data quality (eg, duplicate measurements, training of assessors) and a description of study instruments (eg, questionnaires, laboratory tests) along with their reliability and validity, if known. Reference to where data collection forms can be found, if not in the protocol
	18b	Plans to promote participant retention and complete follow-up, including list of any outcome data to be collected for participants who discontinue or deviate from intervention protocols
Data management	19	Plans for data entry, coding, security, and storage, including any related processes to promote data quality (eg, double data entry; range checks for data values). Reference to where details of data management procedures can be found, if not in the protocol
Statistical methods	20a	Statistical methods for analysing primary and secondary outcomes. Reference to where other details of the statistical analysis plan can be found, if not in the protocol
	20b	Methods for any additional analyses (eg, subgroup and adjusted analyses)
	20c	Definition of analysis population relating to protocol non-adherence (eg, as randomised analysis), and any statistical methods to handle missing data (eg, multiple imputation)
**Methods: Monitoring**		
Data monitoring	21a	Composition of data monitoring committee (DMC); summary of its role and reporting structure; statement of whether it is independent from the sponsor and competing interests; and reference to where further details about its charter can be found, if not in the protocol. Alternatively, an explanation of why a DMC is not needed

	21b	Description of any interim analyses and stopping guidelines, including who will have access to these interim results and make the final decision to terminate the trial
Harms	22	Plans for collecting, assessing, reporting, and managing solicited and spontaneously reported adverse events and other unintended effects of trial interventions or trial conduct
Auditing	23	Frequency and procedures for auditing trial conduct, if any, and whether the process will be independent from investigators and the sponsor
**Ethics and dissemination**		
Research ethics approval	24	Plans for seeking research ethics committee/institutional review board (REC/IRB) approval
Protocol amendments	25	Plans for communicating important protocol modifications (eg, changes to eligibility criteria, outcomes, analyses) to relevant parties (eg, investigators, REC/IRBs, trial participants, trial registries, journals, regulators)
Consent or assent	26a	Who will obtain informed consent or assent from potential trial participants or authorised surrogates, and how (see Item 32)
	26b	Additional consent provisions for collection and use of participant data and biological specimens in ancillary studies, if applicable
Confidentiality	27	How personal information about potential and enrolled participants will be collected, shared, and maintained in order to protect confidentiality before, during, and after the trial
Declaration of interests	28	Financial and other competing interests for principal investigators for the overall trial and each study site
Access to data	29	Statement of who will have access to the final trial dataset, and disclosure of contractual agreements that limit such access for investigators
Ancillary and post-trial care	30	Provisions, if any, for ancillary and post-trial care, and for compensation to those who suffer harm from trial participation
Dissemination policy	31a	Plans for investigators and sponsor to communicate trial results to participants, healthcare professionals, the public, and other relevant groups (eg, via publication, reporting in results databases, or other data sharing arrangements), including any publication restrictions
	31b	Authorship eligibility guidelines and any intended use of professional writers
	31c	Plans, if any, for granting public access to the full protocol, participant-level dataset, and statistical code

For peer review

Appendices

Informed consent materials	32	Model consent form and other related documentation given to participants and authorised surrogates
Biological specimens	33	Plans for collection, laboratory evaluation, and storage of biological specimens for genetic or molecular analysis in the current trial and for future use in ancillary studies, if applicable

*It is strongly recommended that this checklist be read in conjunction with the SPIRIT 2013 Explanation & Elaboration for important clarification on the items. Amendments to the protocol should be tracked and dated. The SPIRIT checklist is copyrighted by the SPIRIT Group under the Creative Commons "Attribution-NonCommercial-NoDerivs 3.0 Unported" license.

For peer review only

BMJ Open

A randomised, placebo-controlled, phase 3 trial of the effect of the omega-3 polyunsaturated fatty acid eicosapentaenoic acid (EPA) on colorectal cancer recurrence and survival after surgery for resectable liver metastases: EPA for Metastasis Trial 2 (EMT2) study protocol

Journal:	*BMJ Open*
Manuscript ID	bmjopen-2023-077427.R1
Article Type:	Protocol
Date Submitted by the Author:	25-Oct-2023
Complete List of Authors:	Hull, Mark; University of Leeds<br>Ow, Pei Loo; University of Leeds, Leeds Institute of Clinical Trials Research<br>Ruddock, Sharon; University of Leeds, Leeds Institute of Clinical Trials Research<br>Brend, Tim; University of Leeds, Leeds Institute of Medical Research<br>Smith, Alexandra; University of Leeds, Leeds Institute of Clinical Trials Research<br>Marshall, Helen; University of Leeds, Leeds Institute of Clinical Trials Research<br>Sang, Mingyang; Harvard University T H Chan School of Public Health<br>Chan, AT; Massachusetts General Hospital<br>Garrett, Wendy; Harvard University T H Chan School of Public Health<br>Yilmaz, Omer; Massachusetts Institute of Technology Koch Institute for Integrative Cancer Research<br>Drew, David; Massachusetts General Hospital<br>Collinson, Fiona; University of Leeds Clinical Trials Research Unit<br>Cockbain, Andrew; Leeds Teaching Hospitals NHS Trust<br>Jones, Robert; Royal Liverpool and Broadgreen University Hospitals NHS Trust<br>Loadman, Paul; University of Bradford, Institute of Cancer Therapeutics<br>Hall, Peter; University of Edinburgh Division of Clinical and Surgical Sciences, Edinburgh Clinical Trials Unit<br>Moriarty, Catherine ; Leeds Teaching Hospitals NHS Trust,<br>Cairns, David; University of Leeds, Leeds Institute of of Clinical Trials Research<br>Toogood, Giles; University of Leeds, Leeds Institute of Medical Research; Leeds Teaching Hospitals NHS Trust
**Primary Subject Heading**:	Oncology
Secondary Subject Heading:	Gastroenterology and hepatology
Keywords:	Gastrointestinal tumours < ONCOLOGY, Hepatobiliary surgery < SURGERY, Clinical trials < THERAPEUTICS

SCHOLARONE™
Manuscripts

A randomised, placebo-controlled, phase 3 trial of the effect of the omega-3 polyunsaturated fatty acid eicosapentaenoic acid (EPA) on colorectal cancer recurrence and survival after surgery for resectable liver metastases: EPA for Metastasis Trial 2 (EMT2) study protocol

Running title: A randomised trial of EPA in patients with colorectal liver metastasis

Mark A Hull[1*]

Pei Loo Ow[2]

Sharon Ruddock[2]

Tim Brend[1]

Alexandra F Smith[2]

Helen Marshall[2]

Mingyang Song[3]

Andrew T Chan[4]

Wendy S Garrett[3]

Omer Yilmaz[5]

David A Drew[4]

Fiona Collinson[2]

Andrew J Cockbain[6]

Robert Jones[7]

Paul M Loadman[8]

Peter S Hall[9]

Catherine Moriarty[6]

David A Cairns[2]

Giles J Toogood[1,6]

[1]Leeds Institute of Medical Research, University of Leeds, Leeds, UK

[2]Leeds Cancer Research UK Clinical Trials Unit, Leeds Institute of Clinical Trials Research, University of Leeds, Leeds, UK

[3]Harvard TH Chan School of Public Health, Boston, USA

For peer review only - http://bmjopen.bmj.com/site/about/guidelines.xhtml

For peer review only

[4]Clinical and Translational Epidemiology Unit, Massachusetts General Hospital, Boston, USA

[5]Koch Institute for Integrative Cancer Research at Massachusetts Institute of Technology, Boston, USA

[6]Leeds Teaching Hospitals NHS Trust, Leeds, UK

[7]Royal Liverpool and Broadgreen University Hospitals NHS Trust, Liverpool, UK

[8]Institute for Cancer Therapeutics, University of Bradford, Bradford, UK

[9]Edinburgh Clinical Trials Unit, University of Edinburgh

*Correspondence to Professor Mark Hull, Leeds Institute of Medical Research, Wellcome Trust Brenner Building, St James's University Hospital, Leeds LS9 7TF, United Kingdom (M.A.Hull@leeds.ac.uk)

For peer review only

Abstract

Introduction: There remains an unmet need for safe and cost-effective adjunctive treatment of advanced colorectal cancer (CRC). The omega-3 polyunsaturated fatty acid eicosapentaenoic acid (EPA) is safe, well-tolerated, and has anti-inflammatory as well as anti-neoplastic properties. A Phase 2 randomised trial of pre-operative EPA free fatty acid 2 g daily in patients undergoing surgery for CRC liver metastasis showed no difference in the primary endpoint (histological tumour proliferation index) compared with placebo. However, the trial demonstrated possible benefit for the pre-specified exploratory endpoint of post-operative disease-free survival. Therefore, we tested the hypothesis that EPA treatment, started before liver resection surgery (and continued post-operatively), improves CRC outcomes in patients with CRC liver metastasis.

Methods and analysis: The EMT2 trial is a randomised, double-blind, placebo-controlled, phase 3 trial of 4g EPA ethyl ester (icosapent ethyl [IPE; Vascepa®]) daily in patients undergoing liver resection surgery for CRC liver metastasis with curative intent. Trial treatment continues for a minimum of two years and maximum of four years, with six-monthly assessments, including Quality of Life outcomes, as well as annual clinical record review after the the trial intervention. The primary endpoint is CRC progression-free survival (PFS). Key secondary endpoints are overall survival, as well as the safety and tolerability of IPE. A minimum 388 participants are estimated to provide 247 CRC progression events during minimum two-year follow-up, allowing detection of a hazard ratio of 0.7 in favour of IPE, with a power of 80% at the 5% (two-sided) level of significance, assuming drop-out of 15%.

Ethics and dissemination: Ethical and Health Research Authority approval was obtained in January 2018. All data will be collected by 2025. Full trial results will be published in 2026. Secondary analyses of health economic data, biomarker studies and other translational work will be published subsequently.

Trial registration number: NCT03428477

For peer review only - http://bmjopen.bmj.com/site/about/guidelines.xhtml

Strengths and limitations of this study

- EMT2 is a randomised, double-blind, placebo-controlled trial of 4 g pure EPA ethyl ester daily
- Treatment starts before CRC liver metastasis surgery and is scheduled for a minimum two years
- The primary endpoint is progression-free survival with a key secondary endpoint of overall survival
- Participant follow-up and IMP delivery can be performed remotely by telephone and courier delivery, which provided COVID-resilience during the Pandemic
- There is a linked biospecimen collection allowing translational studies investigating the mechanism(s) of the anti-CRC activity of EPA

For peer review only - http://bmjopen.bmj.com/site/about/guidelines.xhtml

Introduction

Colorectal cancer (CRC) remains the second most common cause of cancer-related death globally with 935,173 deaths registered in 2020, which represents 9.4% of all cancer deaths worldwide [1]. Only 53% of individuals diagnosed with CRC in England (2013-2017) survive for ten or more years, with the majority of deaths being related to distant metastasis [2]. Approximately half of all CRC patients present with synchronous or metachronous liver metastasis (LM) [3]. Historically, survival of patients with untreated CRCLM was only a few months [4]. Five-year survival rates for CRCLM patients with contemporary multi-modality treatments are now widely of the order of 30% [5]. Surgical resection provides the only prospect of 'cure' for individuals with CRCLM, who are deemed suitable for surgery, with approximately 20% of those undergoing surgery obtaining long-term (>10 years) disease-free survival [6]. However, overall five-year survival following liver resection with curative intent and oxaliplatin-based adjuvant chemotherapy is, at best, 60% [2]. Therefore, there remains an unmet need for safe and cost-effective adjunctive treatment for patients with CRCLM.

The omega-3 polyunsaturated fatty acid (PUFA) eicosapentaenoic acid (EPA) is found naturally in highest quantities in oily fish [7]. It is widely used as a nutritional supplement, most often in combination with the other main marine omega-3 PUFA docosahexaenoic acid (DHA) [7]. Pure EPA ethyl ester is now licensed for use in patients with severe hypertriglyceridaemia and for secondary prophylaxis of vascular events in high-risk individuals taking a statin, who have elevated serum triglyceride levels [8].

Clinical evidence that EPA has anti-CRC activity at early stages of colorectal carcinogenesis is emerging. Treatment with 99% EPA free fatty acid 2 g daily for 6 months was associated with a significant reduction in rectal adenomatous polyp (the benign precursor of CRC) size (29.8%) and multiplicity (22.4%) compared with placebo in a randomised controlled trial (RCT) in patients with familial adenomatous polyposis undergoing sigmoidoscopic surveillance after total colectomy [9]. The seAFOod polyp prevention trial reported that treatment with EPA free fatty acid 2 g daily was associated with a reduction in adenomatous (but not serrated) colorectal polyp recurrence (as measured by colorectal polyp number) in 'high risk' individuals undergoing colonoscopy surveillance in the English Bowel Cancer Screening Programme [10].

Omega-3 PUFAs may also modify the natural history of established CRC. Observational data from cohort studies suggest that increased fish intake is associated with improved survival post-CRC diagnosis, the effect being most prominent for proximal (right-sided) CRCs [11]. The

For peer review only - http://bmjopen.bmj.com/site/about/guidelines.xhtml

EMT study was a phase 2 RCT of EPA free fatty acid 2 g daily in patients awaiting liver resection surgery for CRC liver metastasis [12]. Although the primary endpoint (the histological CRC tumour cell proliferation index) was null, liver metastases in participants from the active EPA arm had a lower vascularity score than placebo-treated tumours, suggesting possible anti-angiogenic activity [12]. In addition, there was an increase in overall survival (OS) and disease-free survival (DFS) in patients randomised to the EPA arm, both of which were specified as exploratory endpoints based on hypotheses that there is prolonged EPA tissue bioavailability due to the slow tissue 'washout' kinetics of EPA and that oral omega-3 PUFA use alters the human gut microbiome [13-14].

There is evidence that concurrent omega-3 PUFA therapy may improve the tolerability and efficacy of cancer chemotherapy from a number of small, heterogeneous studies across several gastrointestinal cancers [15]. Two uncontrolled case series support improved tolerability and nutritional parameters in omega-3 PUFA users during FOLFOX and FOLFIRI regimens for CRC [16-17]. Moreover, a study of omega-3 PUFA-containing oral nutritional supplement (ONS) use demonstrated survival benefit in patients with advanced gastrointestinal cancer receiving cancer chemotherapy and a Glasgow score ≥1 compared with individuals who received no ONS [18]. Systematic review and meta-analysis of the effect of omega-3 PUFA-containing ONS intake on cancer cachexia has highlighted the paucity of high-quality evidence but demonstrated a signal towards anti-inflammatory activity and body weight maintenance for omega-3 PUFA supplementation [19]. Therefore, a valid hypothesis is that EPA treatment abrogates the CRC-related cachexia syndrome characterised by fatigue, anorexia and reduced skeletal muscle mass (sarcopaenia).

In summary, given the promising preliminary data supporting anti-cancer activity of EPA [20], including possible survival benefit for patients with CRCLM [12], and the excellent safety and tolerability profile of EPA [8], we tested the hypothesis that oral treatment with high-dose (4 g daily) pure EPA ethyl ester, started before surgery and continued long-term after liver resection for CRCLM, decreases CRC recurrence, and/or improves overall survival and quality of life, in patients with advanced CRC.

For peer review only - http://bmjopen.bmj.com/site/about/guidelines.xhtml

For peer review only

Trial Design

The EMT2 (**E**PA for **M**etastasis **T**rial **2**) trial is a randomised, double-blind, placebo-controlled, multi-centre, parallel-group, superiority phase 3 trial of EPA in patients undergoing liver resection surgery for CRCLM with curative intent. The trial is approved by Newcastle and North Tyneside Research Ethics Committee (16/NE/0140). The trial is based in tertiary-referral Hepatobiliary Surgery Units in England and Wales. Patients are randomised, on an equal basis, to receive either four soft-gel capsules per day each containing 1 g icosapent ethyl (IPE; Vascepa® [USA] or Vazkepa® [UK and Europe], Amarin Pharma Inc., Bridgewater, NJ, USA), taken as two capsules twice daily with food, or four identical placebo capsules (containing pharmaceutical grade mineral oil [21]) taken in an identical fashion, before liver surgery (Figure 1). The IMP is kindly provided by Amarin Pharma, Inc. and is identical to that used in the REDUCE-IT trial [8] with a daily dose of EPA equivalent to 4 g of EPA-ethyl ester or 3656 mg of EPA free fatty acid). This daily dose of IPE is licensed for use in the USA, UK and Europe for cardiovascular event risk reduction in adult statin-treated patients at high cardiovascular risk with elevated triglycerides (≥ 150 mg/dl [≥ 1.7 mmol/l]) and either established cardiovascular disease, or diabetes and at least one other cardiovascular risk factor. The use of mineral oil as a clinical trial placebo has been reviewed [21]. In the REDUCE-IT trial, 4 g daily of pharmaceutical grade mineral oil was associated with a small increase in levels of inflammatory and lipid biomarkers associated with atherosclerosis risk [22], believed to account for, at most, 3% increased cardiovascular risk in the placebo arm of REDUCE-IT [23]. Overall, there is no evidence that mineral oil, at quantities used in clinical trials including EMT2 and REDUCE-IT, impacts significantly on drug absorption or other clinical outcomes in cardiovascular trials [21].

All participants are scheduled to take Investigational Medicinal Product (IMP) for a minimum of two years and a maximum of four years after liver resection, with continuing annual clinical record review after the end of the intervention phase (Figure 2).

Inclusion and exclusion criteria are listed in Table 1. Patients with synchronous primary and liver metastatic CRC are eligible with recruitment possible at the time of the clinical decision to perform CRCLM surgery. Administration of neo-adjuvant or adjuvant chemotherapy is not an exclusion criterion. Exclusion criteria are also consistent with excipients in the IMP capsule. Consistent with the Summary of Product Characteristics (SmPC) for IPE [24], concurrent use of anti-coagulant or anti-platelet medication is allowed.

For peer review only - http://bmjopen.bmj.com/site/about/guidelines.xhtml

The eligibility of individuals, who are participating in another trial, is determined on a case-by-case basis by the research site team, Clinical Trials Unit (CTU) and Sponsor. Co-enrolment with participants in the CRC cohort of the Add-Aspirin trial (ISRCTN74358648) is permitted providing that the respective eligibility criteria of each trial are fulfilled [25].

The current protocol is version 9.0 dated 27th July 2022. The trial website is https://ctru.leeds.ac.uk/EMT2/.

Trial endpoints

Primary endpoint

The primary endpoint is progression-free survival (PFS) during a minimum of 2 years follow up. Progression-free survival is defined as 1) the time from randomisation to death (from any cause), 2) the first documented evidence of CRC progression (defined as the date of the CT scan or the relevant assessment, at which disease progression or new recurrence is identified and which can be clinical progression or radiological progression evaluated by RECIST1.1 [26]), or 3) new CRC recurrence or clinical deterioration unequivocally due to CRC progression. Participants without a CRC progression event will be censored at the time of the last assessment when they were alive and progression-free.

Secondary and exploratory endpoints

The key secondary endpoint is overall survival (OS), which is defined as the time from randomisation to death from any cause. Participants alive at analysis will be censored at the time of the last assessment when they were alive.

Other secondary endpoints are 1) the safety and tolerability of IPE 4 g daily before and after surgery, including during cancer chemotherapy; 2) patient-reported quality of life measures; 3) cost-effectiveness of IPE assessed using a modified UK Cancer Costs Questionnaire Version 2.0; and 4) new primary cancer (excluding non-melanoma skin cancer, breast ductal cancer *in situ*, cervical carcinoma *in situ*, and superficial bladder carcinoma).

An exploratory endpoint is the red blood cell (RBC) membrane EPA level measured at baseline (visit 1), pre-surgery (visit 2) and 6 months after CRCLM surgery (visit 3) from blood samples taken from two Yorkshire research sites (Leeds and Sheffield) [10]. In addition, the skeletal muscle area at the L3 level on CT imaging at 6 months after CRCLM may be measured as another exploratory endpoint dependent on the availability of additional, specific funding for that analysis.

For peer review only - http://bmjopen.bmj.com/site/about/guidelines.xhtml

Trial schedule

Potential participants are identified by the Hepatobiliary Multi-disciplinary team (MDT) at the time of the decision to offer CRCLM surgery with curative intent (Figure 1). Potential participants who require a primary CRC resection or 'downsizing' neo-adjuvant chemotherapy for CRCLM prior to surgery are reviewed by the MDT again, following primary management, in order to confirmation up-to-date eligibility. A woman of childbearing potential (WOCBP) is required to undergo a pregnancy test prior to randomisation.

After written informed consent is obtained, participants are allocated 1:1, using minimisation with a random element, to active or placebo treatment up to the day before CRCLM surgery using an automated 24-hour telephone/online randomisation service based in Leeds CTU. Minimisation is for 1) neo-adjuvant chemotherapy prior to liver resection; 2) known extrahepatic CRC metastases with curative treatment planned; 3) current regular aspirin use or Add-aspirin trial participant still taking IMP; 4) research site; and 5) time between randomisation and date of planned liver resection surgery either <2 weeks or ≥2 weeks.

The schedule of trial assessments before CRCLM surgery, in the peri-operative period, and post-operatively is described in Figure 2 and Supplementary Figure 1. Assessments are carried out every two weeks between randomisation and CRCLM surgery by telephone and on the first day of the hospital stay for CRCLM surgery. Post-operative assessments are six-monthly with a minimum CT Thorax/Abdomen/Pelvis every 6 months for two years followed by annual CT imaging thereafter, in line with routine clinical practice at most sites. Quality of life (QoL) is assessed using EQ-5D-5L, EORTC QLQ-C30 and QLQ-LMC21 supplementary module questionnaires. Health economic outcomes are assessed using a modified UK Cancer Costs Questionnaire Version 2.0 at each trial visit and telephone call.

Participants are followed up for 60 days beyond the end of IMP treatment, followed by annual case record review in order to collect CRC progression and OS data.

Sample size

Prior to the trial opening, 448 participants (224 per arm) were anticipated to give 247 CRC progression events that are required to detect a hazard ratio (HR) of 0.7 in favour of the treatment arm with a power of 80% at the 5% (two-sided) level of significance. The sample size estimate assumed that the control arm would have a median PFS of 21 months and the

For peer review only - http://bmjopen.bmj.com/site/about/guidelines.xhtml

treatment arm would have a median PFS of 30 months [27-28], with recruitment in a two-year period, minimum two-year follow-up per participant and with 10% drop-out.

The HR for PFS in the Phase II EMT study was 0.694 [12], but the EPA arm, by chance, recruited a higher proportion of patients with poor prognostic features, and a multivariate analysis including these factors actually yielded a HR of 0.35 (95% confidence interval: 0.15-0.79) lending credence to the assumed more conservative HR of 0.7 chosen for this larger phase 3 study, given that the magnitude of benefit observed in phase 2 studies is often greater than that observed in the phase III counterpart.

The EMT study tested EPA-FFA 2g daily [12]. Although the EMT2 study is evaluating IPE 4 g daily, we have assumed at least similar pharmacokinetics of EPA given as IPE, compared with lower-dose EPA-FFA.

Slower than expected recruitment and a higher withdrawal rate due to the COVID-19 pandemic and other factors (see *Trial Progress*) prompted re-evaluation of the sample size required to generate 247 CRC progression events during an extended recruitment period (5.5 years), which provides longer cumulative follow-up. We used a generalized F distribution, rather than exponential function, which more accurately reflected blinded, live PFS data. A simulation study (1000 simulations with random selection of 90% of participants to account for drop-out) in October 2021 predicted that a minimum 388 participants would be required to observe 247 CRC progression events (accounting for actual participant withdrawals, including those related to a pause of the trial at one research site during the first COVID-19 lockdown).

Treatment

Participants receive either four 1 g capsules of IPE or four identical placebo capsules daily (two capsules twice daily with food) from the date of randomisation until the participant has completed study treatment. Treatment is scheduled to continue after disease progression or diagnosis of a new primary cancer. Treatment compliance is assessed at each trial visit or telephone call to determine if the participant has delayed, missed or modified dosing. Unused, expired capsules are collected from participants at a trial visit or at the end of the scheduled intervention period, for counting.

A participant is allowed to take one or more treatment breaks during the trial follow-up, if the participant experiences a persistent adverse reaction (AR), or difficulty taking IMP (for

For peer review only - http://bmjopen.bmj.com/site/about/guidelines.xhtml

example, because of restrictions to oral intake, during CRC chemotherapy, or due to other concurrent treatment). In the event of a persistent AR, dose reduction (to two capsules daily) is recommended prior to a complete trial treatment break.

'Over-the-counter' nutritional supplements containing any omega-3 PUFA or prescribed omega-3 PUFAs (eg. Omacor®) are prohibited while participants are receiving trial treatment and are an exclusion criterion for the EMT2 trial. Any participant consistently using another omega-3 PUFA preparation is withdrawn from trial treatment. Concomitant use of aspirin, other anti-platelet agents, or anticoagulants such as warfarin or a direct-acting oral anticoagulant (DOAC) are allowed, in keeping with the SmPC for IPE (Vazkepa®) [24].

Safety

Reference Safety Information with which to assess seriousness, expectedness and causality for adverse events (AEs) was an Investigator Brochure for IPE (Vascepa®) version 2 dated 1/6/2022 until January 2022, after which the SmPC for IPE (Vazkepa®) [24] was used after the MHRA granted a licence for use of IPE for secondary prophylaxis of vascular events in high-risk individuals taking a statin, who have elevated serum triglyceride levels.

Statistical analysis

A full statistical analysis plan will be written before any analysis is undertaken. Analysis will be conducted on an intention-to-treat basis, in which participants will be included according to the treatment they were randomised to receive, apart from safety/tolerability endpoints which will be based on a safety population of all patients who took at least one dose of any trial treatment.

All hypothesis tests will be two-sided and will use a 5% significance level. No interim futility or early stopping analyses are planned. The final analysis will only take place after the last patient recruited has been followed up for a minimum 24 months, has withdrawn, or died.
The primary endpoint (PFS) and the key secondary endpoint (OS) will reported using the 95% confidence interval of the HR. PFS and OS curves will be calculated using the Kaplan-Meier method. Differences in PFS and OS between the treatment groups will be compared using multivariate modelling to adjust for the minimisation factors. Treatment HRs and corresponding 95% confidence intervals will be obtained from the multivariate models.

For peer review only - http://bmjopen.bmj.com/site/about/guidelines.xhtml

A subgroup analysis comparing the outcomes for patients that receive ≥2 weeks of IMP prior to surgery and patients that receive <2 weeks of EPA will be performed. Sensitivity analyses for PFS and OS will be conducted to examine the effects of other potential modifiers, in addition to the minimisation factors. Other sensitivity analyses may be carried out for each endpoint to take into account differing assumptions about missing data, if there is a significant amount of missing data. Further subgroup and sensitivity analyses may also be performed and will be detailed in the full statistical analysis plan.

Safety information will be summarised by treatment received. The suspected relationship to IPE will be presented along with other causality, the outcome and the event duration. The number and timing of new primary cancers will also be summarised descriptively by treatment group.

QoL measures will be analysed using random effects (multi-level) models to account for the hierarchical nature of repeated measures data and the models will include adjustments for baseline QoL and the minimisation factors. QoL will be presented using mean scores along with the 95% confidence interval. Similar summaries will be produced for quality-adjusted life years (QALYs), as scored by the EQ-5D-5L questionnaire. Cost-effectiveness comparisons between treatment arms will be analysed using the incremental cost effectiveness ratio. The cost per disease recurrence prevented at 2 years and the cost per QALY at 2 years will be reported.

The relationship between the RBC membrane EPA level and both PFS and OS will be analysed using an extended Cox model, which will adjust for the minimisation factors and will also include the RBC membrane EPA level (% of total fatty acids) [10] as a time-dependent covariate.

For peer review only

Trial progress

Health Research Authority approval (Newcastle and North Tyneside REC 16/NE/0140 and MHRA CTA 16767/0289/) was granted on 3/4/2017. EMT2 was prospectively registered with ClinicalTrials.gov (NCT03428477). The European Clinical Trials Database registration is EudraCT 2016-000628-24.

The EMT2 trial opened to recruitment in March 2018. First Participant First Visit was 2/5/2018. The original recruitment period was estimated to be 24 months based on a recruitment projection of 18 patients per month by 8 research sites. This recruitment estimate

For peer review only - http://bmjopen.bmj.com/site/about/guidelines.xhtml

was based on the annual CRCLM surgery volume at the research sites in 2014 (890 procedures) and a 25% randomisation rate of screened patients. However, the mean monthly recruitment in the first twelve months was four per month. In the early phase of recruitment, 38% of potential participants undergoing screening were ineligible because CRCLM surgery was scheduled within two weeks of randomisation. This prompted a substantial protocol amendment to allow recruitment of patients without stipulation about the length of IMP treatment before surgery (Supplementary Data).

The number of research sites has since been expanded to 13 (See Supplementary Data for the list of research sites and their date of opening for recruitment).

The EMT2 trial did not recruit any participants for approximately two months at the onset of the COVID-19 pandemic restrictions in the UK in early 2020, although, in contrast with many randomised trials in the UK, the trial remained open, except for one NHS Trust research site that paused all non-COVID-19 research activity for several months from March 2020. During the COVID-19 pandemic, recruitment continued with wide variations in monthly recruitment rates related to research staff availability (affected by secondment to COVID-19 research roles and sickness), alterations to routine Hepatobiliary Surgery activity and practice, and patient acceptability. The ability to deliver IMP by post and perform trial follow-up visits by telephone aided trial acceptability and retention during the pandemic. We did not observe an increase in trial withdrawal during national COVID-19 lockdowns between March 2020 and mid-2022.

Based on a revised recruitment projection of 5 participants per month, in order to reach a revised recruitment target of 388, the recruitment period has been extended to 67 months (end of recruitment November 2023) with a minimum two-year follow-up. In order to collect the maximum number of CRC progression events from randomised individuals, the protocol was amended to obtain consent from participants at the end of their four-year intervention period to collect data on any CRC progression event from their healthcare record on an annual basis until the end of the follow-up period of the EMT2 trial at the end of November 2025.

Other Protocol amendments during EMT2 trial conduct are listed in Supplementary Data. Key changes during the trial were removal of the stipulation that IMP was started more than two weeks before CRCLM surgery (prompted by realisation that the majority of surgeries were performed within two weeks of the decision to offer CRCLM resection; SA06), and addition of new exclusion criteria related to peanut/soya allergy and sorbitol/xylitol intolerance (SA14).

For peer review only - http://bmjopen.bmj.com/site/about/guidelines.xhtml

EMT2 Biospecimen collection and ancillary translational studies

Separate funding was obtained to collect faeces, urine, blood and tumour tissue from EMT2 participants after randomisation to the EMT2 trial for translational laboratory studies. This work is performed under separate HRA approval (REC 20/YH/0306) and ClinicalTrials.gov registration (NCT04682665) as the EMT2 Biospecimen collection. The current protocol for Biospecimen collection is version 3.0 dated 28th April 2022.

The EMT2 biospecimen collection is part of a collaboration between the University of Leeds and researchers across several institutions in Boston, USA (Massachussetts General Hospital [MGH], Harvard TH Chan School of Public Health [HSPH] and Massachussetts Institute of Technology [MIT]) and the University of Bradford, funded by National Institutes of Health to investigate the role of the gut microbiota and its relationship with the host anti-tumour immune response in the anti-CRC activity of EPA.

Consent to provide biospecimens is obtained from new EMT2 participants, only after EMT2 entry. Faecal, blood and urine samples are collected 1) after EMT2 trial randomisation, before starting active EPA or placebo, 2) just before surgery, and 3) at 6-months after CRCLM surgery (Supplementary Figure 2), with the option to provide further specimens during EMT2 follow-up out to four years. It is also possible for existing EMT2 participants to provide a 'one-off' faecal and urine sample at any time whilst taking IMP during the follow-up period. There are five participating sites, from which baseline (visit 1) and surgery (visit 2) samples are transferred to the Biobank in Leeds. We use a modified Micro-N collection tool [29], which facilitates collection of faecal specimens in a DNA Genotek Inc. OMNIgene.GUT tube (for metagenome and metatranscriptome profiling), in 95% ethanol (for faecal metabolomic analysis), and an anaerobic tube filled anaerobically with liquid dental transport medium (for culture and gnotobiotic animal model studies), for postal delivery to the Leeds Biobank, alongside a urine sample (Supplementary Figure 2).

Laboratory studies will take place in Boston, Leeds and Bradford (Supplementary Figure 3). Linked, fully anonymised data (including EMT2 survival outcomes) will be shared between Leeds and MGH using the EMT2 trial Safety Statistician as the 'honest broker' in order to avoid inadvertent disclosure of individual treatment allocation to EMT2 Investigators (Supplementary Figure 3).

Patient and Public Involvement

For peer review only - http://bmjopen.bmj.com/site/about/guidelines.xhtml

A Patient representative was involved in trial design and is a member of the Trial Management Group. This Patient representative has supported design of all patient-facing materials including website content and Instructions for Biospecimen collection. A different Patient representative sits on the Trial Steering Committee.

For peer review only

For peer review only - http://bmjopen.bmj.com/site/about/guidelines.xhtml

References

1. Ferlay J, Ervik M, Lam F, Colombet M, Mery L, Piñeros M, Znaor A, Soerjomataram I, Bray F (2020). Global Cancer Observatory: Cancer Today. Lyon, France: International Agency for Research on Cancer. Available from: https://gco.iarc.fr/today, accessed [21 June 2023].
2. Cancer Research UK CancerStats. http://www.cancerresearchuk.org/cancer-info/cancerstats/types/bowel/ (accessed 21 June 2023).
3. Hackl C, Neumann P, Gerken M, Loss M, Klinkhammer-Schalke M, Schlitt HJ. Treatment of colorectal liver metastases in Germany: a ten-year population-based analysis of 5772 cases of primary colorectal adenocarcinoma. BMC Cancer 2014;14:810
4. Bengtsson G, Carlsson G, Hafström L, Jönsson PE. Natural history of patients with untreated liver metastases from colorectal cancer. Am J Surg 1981;141:586-589.
5. Roberts KJ, White A, Cockbain A, *et al*. Performance of prognostic scores in predicting long-term outcome following resection of colorectal liver metastases. Br J Surg 2014;101:856-866.
6. Creasy JM, Sadot E, Koerkamp BG, Chou JF, Gonen M, Kemeny NE, Balachandran VP, Kingham TP, DeMatteo RP, Allen PJ, Blumgart LH, Jarnagin WR, D'Angelica MI. Actual 10-year survival after hepatic resection of colorectal liver metastases: what factors preclude cure? Surgery. 2018;163:1238-1244.
7. Cholewski M, Tomczykowa M, Tomczyk M. A Comprehensive Review of Chemistry, Sources and Bioavailability of Omega-3 Fatty Acids. Nutrients 2018;10:1662.
8. Bhatt DL, Steg PG, Miller M, *et al*. Cardiovascular risk reduction with icosapent ethyl for hypertriglceridemia. N Engl J Med 2019;380:11-22.
9. West NJ, Clark SK, Phillips RKS, *et al*. Eicosapentaenoic acid reduces rectal polyp number and size in familial adenomatous polyposis. Gut 2010;59:918-925.
10. Hull MA, Sprange K, Hepburn T, *et al*. Eicosapentaenoic acid and aspirin, alone and in combination, for the prevention of colorectal adenomas (seAFOod Polyp Prevention trial): a multicentre, randomised, double-blind, placebo-controlled, 2 × 2 factorial trial. Lancet 2018;392:2583-2594.
11. Song M, Chan AT. Environmental Factors, Gut Microbiota, and Colorectal Cancer Prevention. Clin Gastroenterol Hepatol 2019;17:275-289.

For peer review only - http://bmjopen.bmj.com/site/about/guidelines.xhtml

12. Cockbain AJ, Volpato M, Race AD, *et al*. Anti-colorectal cancer activity of the omega-3 polyunsaturated fatty acid eicosapentaenoic acid. Gut 2014;63:1760-1768.
13. Cao J, Schwichtenberg KA, Hanson NQ, *et al*. Incorporation and clearance of omega-3 fatty acids in erythrocyte membranes and plasma phospholipids. Clin Chem 2006;52:2265-2272.
14. Watson H, Mitra S, Croden FC, *et al*. A randomised trial of the effect of omega-3 polyunsaturated fatty acid supplements on the human intestinal microbiota. Gut 2018;67:1974-1983.
15. de van der Schueren MAE, Laviano A, Blanchard H, *et al*. Systematic review and meta-analysis of the evidence for oral nutritional intervention on nutritional and clinical outcomes during chemo(radio)therapy: current evidence and guidance for design of future trials. Ann Oncol 2018;29:1141-1153.
16. Trabal J, Leyes P, Forga M, *et al*. Potential usefulness of an EPA-enriched nutritional supplement on chemotherapy tolerability in cancer patients without overt malnutrition. Nutr Hosp 2010;25:736-740.
17. Read JA, Beale PJ, Volker DH, *et al*. Nutrition intervention using an eicosapentaenoic acid (EPA)-containing supplement in patients with advanced colorectal cancer. Effects on nutritional and inflammatory status: a phase II trial. Support Care Cancer 2007;15:301-307.
18. Shirai Y, Okugawa Y, Hishida A, *et al*. Fish oil-enriched nutrition combined with systemic chemotherapy for gastrointestinal cancer patients with cancer cachexia. Sci Rep 2017;7:4826.
19. de Castro GS, Andrade MF, Pinto FCS, *et al*. Omega-3 fatty acid supplementation and its impact on systemic inflammation and body weight in patients with cancer cachexia – A systematic review and meta-analysis. Front Nutr 2022;8:797513.
20. Aldoori J, Cockbain AJ, Toogood GJ, *et al*. Omega-3 polyunsaturated fatty acids: moving towards precision use for prevention and treatment of colorectal cancer. Gut 2022;71:822-837.
21. Olshansky B, Chung MK, Budoff MJ, *et al*. Mineral oil: safety and use as placebo in REDUCE-IT and other clinical studies. Eur Heart J Suppl 2020;22:J34–J48.
22. Ridker PM, Rifai N, MacFadyen J, *et al*. Effects of Randomized Treatment With Icosapent Ethyl and a Mineral Oil Comparator on Interleukin-1β, Interleukin-6, C-

For peer review only - http://bmjopen.bmj.com/site/about/guidelines.xhtml

Reactive Protein, Oxidized Low-Density Lipoprotein Cholesterol, Homocysteine, Lipoprotein(a), and Lipoprotein-Associated Phospholipase A2: A REDUCE-IT Biomarker Substudy. Circulation 2022;146:372-379.

23. Sherratt SCR. REDUCE-IT, biomarkers, and confirmation bias: are we missing the forest for the trees? Eur J Prev Cardiol 2023; doi: 10.1093/eurjpc/zwad169.
24. Summary of Product Characteristics for Icosapent Ethyl (Vazkepa®). https://www.medicines.org.uk/emc/product/12964
25. Coyle C, Cafferty FH, Rowley S, *et al*. ADD-ASPIRIN: A phase III, double-blind, placebo controlled, randomised trial assessing the effects of aspirin on disease recurrence and survival after primary therapy in common non-metastatic solid tumours. Contemp Clin Trials 2016;51:56-64.
26. Eisenhauer EA, Therasse P, Bogaerts J, *et al.* New response criteria in solid tumours: revised RECIST guideline (version 1.1). Eur J Cancer 2009;45:228-247.
27. Nordlinger B, Sorbye H, Glimelius B, *et al*. Perioperative chemotherapy with FOLFOX4 and surgery versus surgery alone for resectable liver metastases from colorectal cancer (EORTC Intergroup trial 40983): a randomised controlled trial. Lancet 2008;371:1007-1016.
28. Primrose J, Falk S, Finch-Jones M, *et al*. Systemic chemotherapy with or without cetuximab in patients with resectable colorectal liver metastasis: the New EPOC randomised controlled trial. Lancet Oncol 2014;15:601-611.
29. Everett C, Li C, Wilkinson JE, *et al.* Overview of the Microbiome Among Nurses study (Micro-N) as an example of prospective characterization of the microbiome within cohort studies. Nat Protoc 2021;16:2724–2731.

For peer review only

For peer review only - http://bmjopen.bmj.com/site/about/guidelines.xhtml

Acknowledgements

The Trial Management Group wish to thank Amarin Pharma, Inc. for providing active and placebo IMP for the EMT2 trial. Amarin Pharma, Inc. played no role in the design or conduct of the EMT2 trial. The support of the Clinical Trials Research Unit (CTRU) at the University of Leeds is essential to the successful running of the study; we thank all CTRU staff who have contributed, past and present. The Trial Management Group also wish to acknowledge the important role played by the Patient & Public Representatives on the Trial Management Group and Trial Steering Committee.

Authors' contributions

MAH is the Chief Investigator. **MAH:** Conceptualization, Investigation, Resources, Writing – Original Draft, Writing – Review & Editing, Supervision, Project Administration, Funding acquisition **PLO:** Methodology, Formal analysis, Investigation, Data Curation, Writing – Original Draft, Writing – Review & Editing **SR:** Investigation, Writing – Review & Editing, Visualization **TB:** Investigation, Resources, Data Curation, Writing – Review & Editing **AFS:** Methodology Investigation, Data Curation, Writing – Review & Editing, Supervision, Project Administration **HM:** Methodology, Formal analysis **MS:** Conceptualization, Resources, Writing – Review & Editing, Funding acquisition **ATC:** Conceptualization, Resources, Writing – Review & Editing, Supervision, Project Administration, Funding acquisition **WSG:** Conceptualization, Investigation, Resources, Writing – Review & Editing, Funding acquisition **OY:** Conceptualization, Investigation, Resources, Writing – Review & Editing, Funding acquisition **DAD:** Investigation, Data Curation **FC:** Conceptualization, Writing – Review & Editing, Funding acquisition **AJC:** Investigation, Resources, Writing – Review & Editing **RJ:** Investigation, Resources, Writing – Review & Editing **PML:** Methodology, Investigation, Writing – Review & Editing, Supervision, Funding acquisition **PSH:** Methodology, Resources, Writing – Review & Editing, Funding acquisition **CM:** Investigation, Writing – Review & Editing **DAC:** Methodology, Formal analysis, Investigation, Data Curation, Writing – Review & Editing, Supervision, Project Administration **GJT:** Conceptualization, Investigation, Resources, Writing – Review & Editing, Supervision, Project Administration, Funding acquisition

For peer review only - http://bmjopen.bmj.com/site/about/guidelines.xhtml

For peer review only

Funding

The EMT2 trial is funded by Yorkshire Cancer Research (L387) and is sponsored by the University of Leeds. The EMT2 Biospecimen collection is funded by the National Institutes of Health (1R01CA243454-01A1) and is sponsored by the University of Leeds (governance-ethics@leeds.ac.uk). Both studies have been adopted to the NIHR Clinical Research Network (CRN) Portfolio (CPMS ID 34700 and 47372, respectively) and have benefited from CRN research staff support.

The funder and the Sponsor had no role in the design of the trial, data collection, or input into the writing of this manuscript.

Competing interests

The Authors declare no Conflict of Interest

Data Availability Statement

A data sharing plan is available in the ClinicalTrials.gov Registry record (NCT NCT03428477).

For peer review only

For peer review only - http://bmjopen.bmj.com/site/about/guidelines.xhtml

Figure Legends

Figure 1. Pathway for screening, obtaining informed consent and randomisation to the EMT2 trial. *the full list of inclusion and exclusion criteria are listed in Table 1. CRC, colorectal cancer; CRCLM, colorectal cancer liver metastasis; IMP, Investigational Medicinal Product; MDT, Multi-disciplinary team meeting; O3FA, omega-3 fatty acid.

Figure 2. Assessment schedule from randomisation onwards in the EMT2 trial. *the EMT2 Biospecimen collection is a distinct study with separate ethical approval and study registration (see text). IMP, Investigational Medicinal Product; IPE, icosapent ethyl.

For peer review only

For peer review only - http://bmjopen.bmj.com/site/about/guidelines.xhtml

Inclusion criteria	Exclusion criteria
Age ≥ 18 years	Previous surgery for the management of current CRCLM†
Able to provide written informed consent	Incurable extra-hepatic metastases
Histological diagnosis of CRC with evidence of one or more liver metastases	Current (in the last two months) or planned regular (more than three doses per week) use of omega-3 PUFA-containing medication or supplements
Planned liver resection surgery for CRCLM with curative intent*	Fish/seafood allergy
Intention to receive IMP treatment prior to CRCLM surgery	hereditary fructose intolerance
	Soya or peanut allergy
	Inability to comply with trial schedule
	Known bleeding tendency (eg. von Willebrand disease)
	A previous malignancy within 5 years‡
	Pregnant/breast-feeding woman or WOCBP not willing to use effective contraceptive measures**
	Fertile (post-pubescent and not permanently sterile by vasectomy or bilateral orchidectomy) man not willing to use barrier contraception

Table 1. Inclusion and exclusion criteria for the EMT2 trial. *includes repeat CRCLM liver surgery (a second independent liver resection) for a separate metachronous CRC liver recurrence. †Patients who have already undergone one CRCLM surgery as part of a two-stage procedure are ineligible. ‡other than CRC, non-melanoma skin cancer where treatment consisted of resection only or radiotherapy, ductal breast carcinoma *in situ* where treatment consisted of resection only, cervical carcinoma *in situ* where treatment consisted of resection

For peer review only - http://bmjopen.bmj.com/site/about/guidelines.xhtml

only, and superficial bladder carcinoma where treatment consisted of resection only. **WOCBP, woman of child-bearing potential: Effective contraception includes combined (oestrogen- and progesterone-containing) hormonal contraception, progesterone-only hormonal contraception, intrauterine device, intrauterine hormone-releasing system, bilateral tubal occlusion, vasectomised partner, practicing true sexual abstinence.

For peer review only

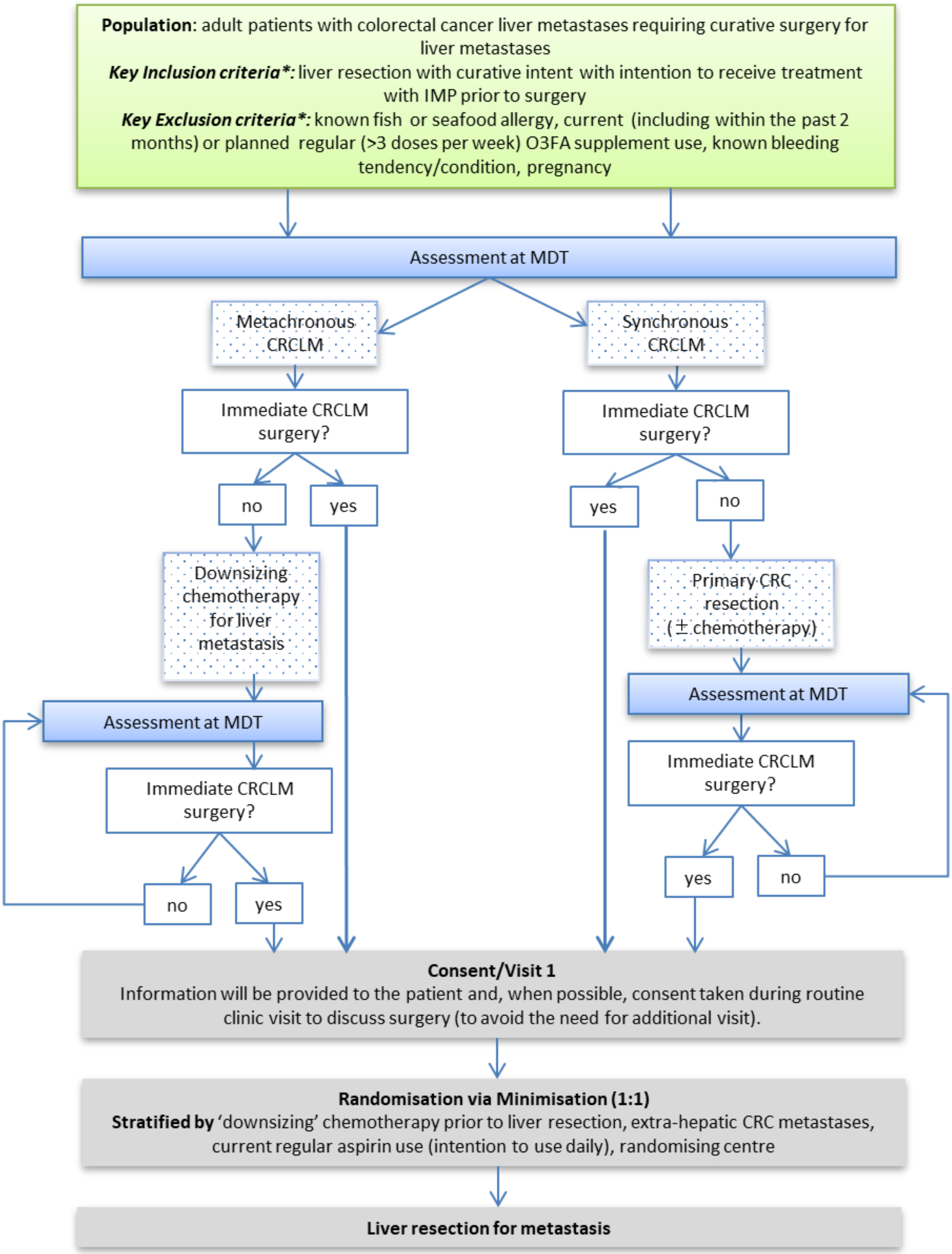
Population: adult patients with colorectal cancer liver metastases requiring curative surgery for liver metastases
Key Inclusion criteria*: liver resection with curative intent with intention to receive treatment with IMP prior to surgery
Key Exclusion criteria*: known fish or seafood allergy, current (including within the past 2 months) or planned regular (>3 doses per week) O3FA supplement use, known bleeding tendency/condition, pregnancy
Assessment at MDT
Metachronous CRCLM
Synchronous CRCLM
Immediate CRCLM surgery?
no
yes
Downsizing chemotherapy for liver metastasis
Assessment at MDT
Immediate CRCLM surgery?
no
yes
Immediate CRCLM surgery?
yes
no
Primary CRC resection (± chemotherapy)
Assessment at MDT
Immediate CRCLM surgery?
yes
no
Consent/Visit 1
Information will be provided to the patient and, when possible, consent taken during routine clinic visit to discuss surgery (to avoid the need for additional visit).
Randomisation via Minimisation (1:1)
Stratified by 'downsizing' chemotherapy prior to liver resection, extra-hepatic CRC metastases, current regular aspirin use (intention to use daily), randomising centre
Liver resection for metastasis

Figure 1

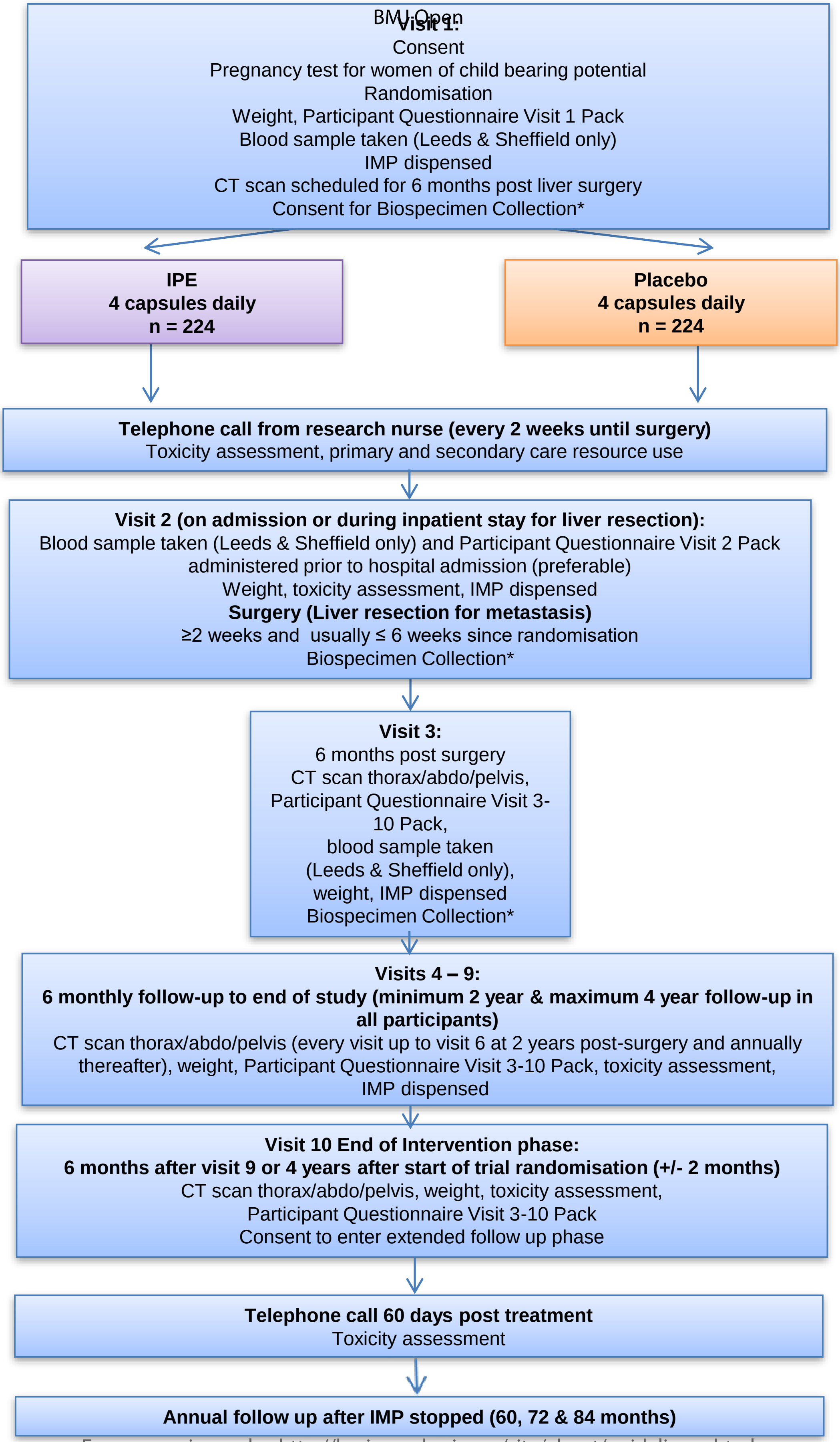
Visit 1:
Consent
Pregnancy test for women of child bearing potential
Randomisation
Weight, Participant Questionnaire Visit 1 Pack
Blood sample taken (Leeds & Sheffield only)
IMP dispensed
CT scan scheduled for 6 months post liver surgery
Consent for Biospecimen Collection*
IPE
4 capsules daily
n = 224
Placebo
4 capsules daily
n = 224
Telephone call from research nurse (every 2 weeks until surgery)
Toxicity assessment, primary and secondary care resource use
Visit 2 (on admission or during inpatient stay for liver resection):
Blood sample taken (Leeds & Sheffield only) and Participant Questionnaire Visit 2 Pack administered prior to hospital admission (preferable)
Weight, toxicity assessment, IMP dispensed
Surgery (Liver resection for metastasis)
≥2 weeks and usually ≤ 6 weeks since randomisation
Biospecimen Collection*
Visit 3:
6 months post surgery
CT scan thorax/abdo/pelvis,
Participant Questionnaire Visit 3-10 Pack,
blood sample taken
(Leeds & Sheffield only),
weight, IMP dispensed
Biospecimen Collection*
Visits 4 – 9:
6 monthly follow-up to end of study (minimum 2 year & maximum 4 year follow-up in all participants)
CT scan thorax/abdo/pelvis (every visit up to visit 6 at 2 years post-surgery and annually thereafter), weight, Participant Questionnaire Visit 3-10 Pack, toxicity assessment, IMP dispensed
Visit 10 End of Intervention phase:
6 months after visit 9 or 4 years after start of trial randomisation (+/- 2 months)
CT scan thorax/abdo/pelvis, weight, toxicity assessment,
Participant Questionnaire Visit 3-10 Pack
Consent to enter extended follow up phase
Telephone call 60 days post treatment
Toxicity assessment
Annual follow up after IMP stopped (60, 72 & 84 months)

Figure 2

A randomised, placebo-controlled, phase 3 trial of the effect of the omega-3 polyunsaturated fatty acid eicosapentaenoic acid (EPA) on colorectal cancer recurrence and survival after surgery for resectable liver metastases: EPA for Metastasis Trial 2 (EMT2) [ClinicalTrials.gov NCT032428477]

Supplementary Data

	Page
1. List of sites recruiting to EMT2	2
2. Substantial Amendments to the EMT2 trial protocol	3
3. Independent members of the Oversight Committees	5

For peer review only

List of sites recruiting to EMT2

Site name	Date open for recruitment
**Basingstoke** (Hampshire Hospitals NHS Foundation Trust)	21/3/2018
**Birmingham** (University Hospitals Birmingham NHS Trust)	20/7/2018
**Cambridge** (Cambridge University Hospitals NHS Foundation Trust)	30/10/2019
**Cardiff** (University Hospital of Wales, Cardiff and Vale University Health Board)	22/6//2020
**Leeds** (Leeds Teaching Hospitals NHS Trust)	9/5/2018
**Liverpool** (Liverpool University Hospitals NHS Foundation Trust)	21/3/2019
**London** (King's College Hospital NHS Foundation Trust)	4/8/2020
**London** (Royal Free London NHS Foundation Trust)	14/10/2019
**Newcastle** (Newcastle upon Tyne Hospitals NHS Foundation Trust)	28/8/2019
**Nottingham** (Nottingham University Hospitals NHS Trust)	5/12/2018
**Oxford** (Oxford University Hospitals NHS Foundation Trust)	29/6/2022
**Sheffield** (Sheffield Teaching Hospitals NHS Foundation Trust)	11/9/2018
**Southampton** (University Hospital Southampton NHS Foundation Trust)	16/3/2018

For peer review only

For peer review only - http://bmjopen.bmj.com/site/about/guidelines.xhtml

Substantial amendments to the EMT2 trial protocol

Substantial amendment (SA)	Protocol version (submission date)	Reason for the amendment[1]
01	2.0 (21/4/2017)	Use of a new IMP[2] (IPE [active] and mineral oil [placebo] capsules) after approval for use of the IMP (90% EPA triglyceride) used in the seAFOod trial[3] was not granted by MHRA.
02	v3.0 (3/6/20217)	Clarifications in relation to pharmacovigilance measures requested by MHRA.
05	v4.0 (23/8/2018)	Addition of 'remote' (telephone) follow-up and IMP delivery for participants who are unable to attend the recruiting centre for in-person assessment.
06	v5.0 (26/2/2019)	Change of eligibility criteria to remove the stipulation that IMP treatment begins more than 2 weeks before liver resection surgery.<br>Addition of exclusion criterion if current liver metastatic disease has already been treated partially by liver resection surgery.<br>Amendment to allow optional dispensing of IMP at visit 2.
12	v6.0 (13/1/2021)	Inclusion of the option for EMT2 participants to join a translational study to provide stool, blood, urine and tumour tissue for laboratory studies (EMT2 Biospecimen study)
14	v7.0 (25/5/2021)	Urgent Safety Measure - amendment to exclude participants with known soya or peanut allergy and add this to the exclusion criteria for trial screening prompted by guidance in the SmPC for Vazkepa™.<br><br>Amendment of the protocol and PISICF to 1) describe atrial fibrillation risk associated with high-dose omega-3 fatty acids and 2) increase emphasis on increased risk of bleeding in individuals on concomitant anti-thrombotic agents.
15	v8.0 (2/11/2021)	Change of the Reference Safety Information from an Investigator Brochure to the SmPC for Vazkepa™.
16	v9.0 (9/9/2022)	Amendment of sample size estimate and recruitment rate projection.<br>Addition of 'remote' follow-up of participant health care records once IMP is stopped.

[1]Ony Substantial Amendments to the Protocol are listed. Other Substantial Amendments were for addition of new research sites, amendment of the PISICF and use of a trial-specific website.

For peer review only - http://bmjopen.bmj.com/site/about/guidelines.xhtml

[2]Abbreviations: EPA, eicosapentaenoic acid; IMP, Investigative Medicinal Product; IPE, icosapent ethyl; MHRA, Medicines and Healthcare Regulatory Agency; PISICF, Patient Information Sheet and Informed Consent Form; SmPC, Summary of Product Characteristics

[3]The IMP used in the latter stages of the seAFOod polyp prevention trial was soft-gel capsules containing 90% EPA triglyceride (active) or capric/caprylic acids (placebo) – see *Trials* 2013;**14**:237.

For peer review only

Independent members of the Oversight Committees

Trial Steering Committee

Professor Richard Wilson (Chair), University of Glasgow

Professor Louise Brown, University College, London

Professor Vicky Coyle, Queen's University, Belfast

Professor Nick Maynard, Oxford University Hospitals NHS Trust

Patient & Public Representative (details available on request with consent)

Yorkshire Cancer Research (Observer)

Data Monitoring Committee

Professor Robert Steele (Chair), University of Dundee

Professor John Norrie, University of Edinburgh

Professor Christopher Byrne, University of Southampton

For peer review only

		Visit 1 (randomisation)	Fortnightly pre-op telephone call	Visit 2: Surgery	Visit 3 (6 months)*	Visit 4 (12 months)	Visit 5 (18 months)	Visit 6 (24 months)	Visit 7 (30 months)	Visit 8 (36 months)	Visit 9 (42 months)	Visit 10 (48 months; End of Intervention phase	60days after treatment end	Extended follow up phase (60, 72 & 84 months)
**Clinical assessments and procedures**	Data Collection from hospital records	x	x	x	x	x	x	x	x	x	x	x	x	x
	CT (thorax/abdomen/pelvis)			x*	x	x	x	x		x		x		
	Body Weight	x		x	x	x	x	x	x	x	x	x		
	Adverse event assessment		x	x	x	x	x	x	x	x	x	x	x	
	Concomitant medications	x	x	x	x	x	x	x	x	x	x	x		
	Compliance monitoring		x	x	x	x	x	x	x	x	x	x		
	Participant Questionnaire Pack	x		x	x	x	x	x	x	x	x	x		
	Pregnancy test	x												
	Blood sample for fatty acid levels	x		x	x									
	Biospecimen collection (optional)	x		x	x	x	x	x	x	x	x	x		
**Trial interventions**	Randomisation	x												
	IMP dispensing	x		x	x	x	x	x	x	x	x			

*In the event that macroscopic tumour remains following liver resection and curative treatment is not possible, a CT scan is carried out as soon as possible to measure the extent of existing disease.

Supplementary Figure 1

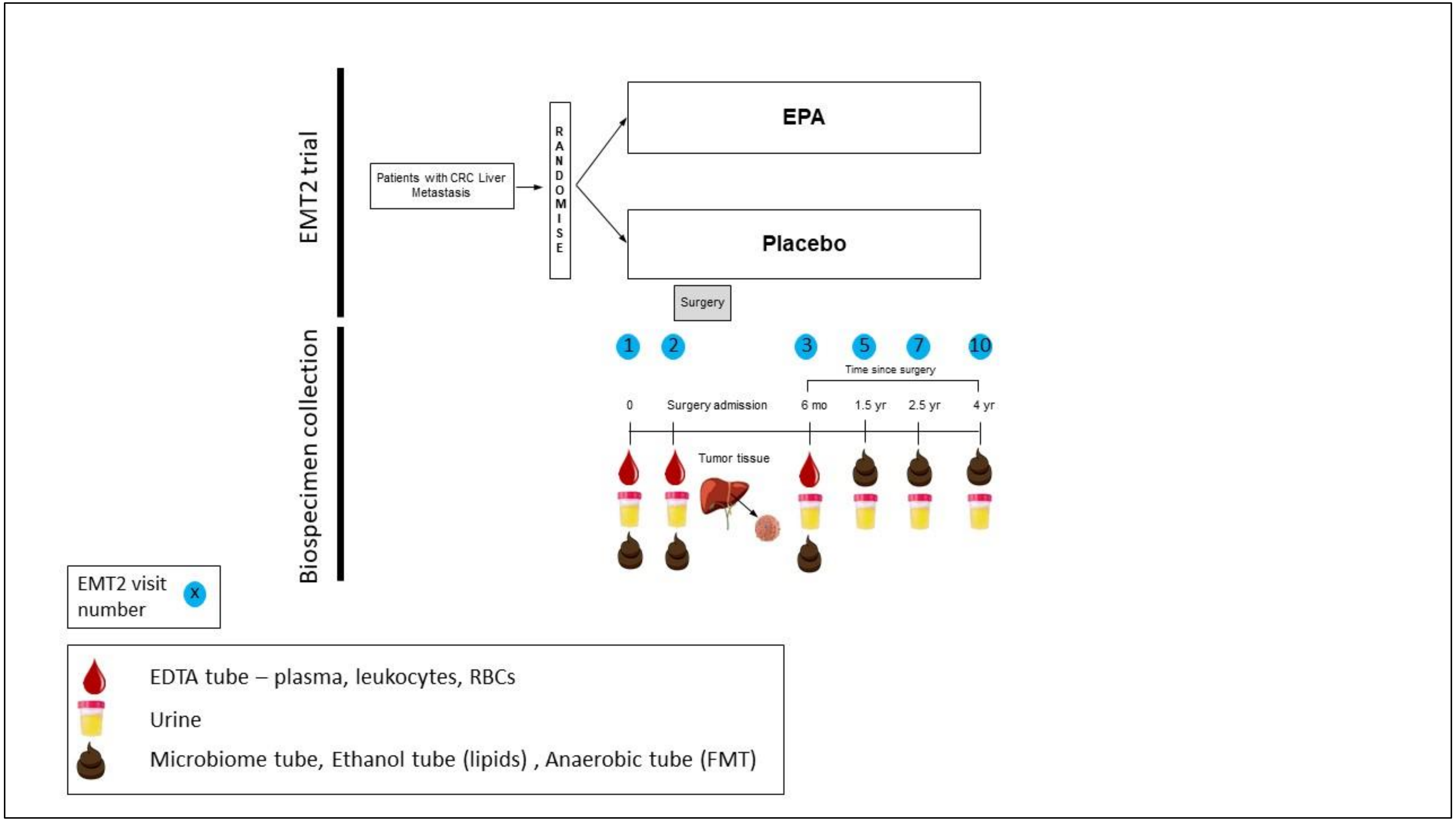

Supplementary Figure 2. Biospecimen collection during EMT2 trial participation. Blood is collected in ethylenediaminetetraacetic acid (EDTA) tubes for immediate fractionation into plasma and red blood cells. A faecal sample is obtained in a DNA Genotek OMNIgeneGUT tube for microbiome analysis, an anaerobic tube for faecal microbial transplant (FMT) studies, and a tube containing 95% ethanol for metabolomic analysis.

For peer review only - http://bmjopen.bmj.com/site/about/guidelines.xhtml

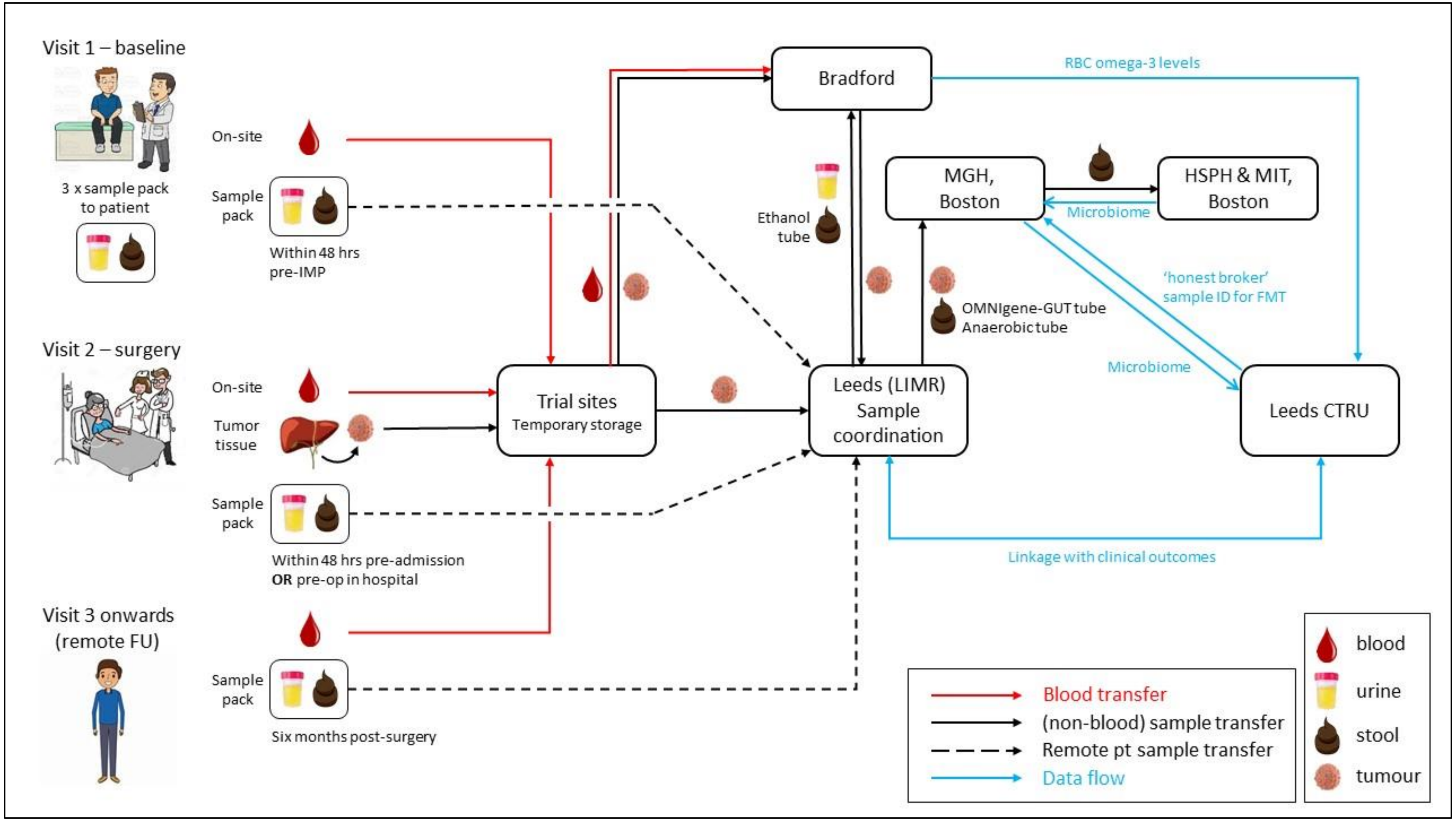

Supplementary Figure 3. Biospecimen and linked data flow during the EMT2 trial. Remote sample transfer from participants to the Leeds Biobank was by Royal Mail Safebox™. Linkage of EMT2 trial outcomes required for design of faecal microbial transplant (FMT) experiments was co-ordinated by the Safety Statistician for EMT2 in order to avoid any disruption of the double-blind for EMT2. CTRU, Clinical Trials Research Unit; HSPH, Harvard School of Public Health; LIMR, Leeds Institute of Medical Research; MGH, Massachussetts General Hospital; MIT, Massachussets Institute of Technology.

STANDARD PROTOCOL ITEMS: RECOMMENDATIONS FOR INTERVENTIONAL TRIALS

SPIRIT 2013 Checklist: Recommended items to address in a clinical trial protocol and related documents*

Section/item	Item No	Description
**Administrative information**		
Title	1	Descriptive title identifying the study design, population, interventions, and, if applicable, trial acronym Page 1
Trial registration	2a	Trial identifier and registry name. If not yet registered, name of intended registry Page 3
	2b	All items from the World Health Organization Trial Registration Data Set
Protocol version	3	Date and version identifier Page 8 Line 1
Funding	4	Sources and types of financial, material, and other support Page 20
Roles and responsibilities	5a	Names, affiliations, and roles of protocol contributors Page 1
	5b	Name and contact information for the trial sponsor Page 20
	5c	Role of study sponsor and funders, if any, in study design; collection, management, analysis, and interpretation of data; writing of the report; and the decision to submit the report for publication, including whether they will have ultimate authority over any of these activities Page 20
	5d	Composition, roles, and responsibilities of the coordinating centre, steering committee, endpoint adjudication committee, data management team, and other individuals or groups overseeing the trial, if applicable (see Item 21a for data monitoring committee) Supplementary data
**Introduction**		
Background and rationale	6a	Description of research question and justification for undertaking the trial, including summary of relevant studies (published and unpublished) examining benefits and harms for each intervention Introduction Page 5-6
	6b	Explanation for choice of comparators Page 7 Line 2
Objectives	7	Specific objectives or hypotheses Page 6 Line 23-28

For peer review only - http://bmjopen.bmj.com/site/about/guidelines.xhtml

Trial design	8	Description of trial design including type of trial (eg, parallel group, crossover, factorial, single group), allocation ratio, and framework (eg, superiority, equivalence, noninferiority, exploratory) Page 7 Line 3

Methods: Participants, interventions, and outcomes

Study setting	9	Description of study settings (eg, community clinic, academic hospital) and list of countries where data will be collected. Reference to where list of study sites can be obtained Page 7 Lines 5-6
Eligibility criteria	10	Inclusion and exclusion criteria for participants. If applicable, eligibility criteria for study centres and individuals who will perform the interventions (eg, surgeons, psychotherapists) Page 7 Line 23 and Table 1
Interventions	11a	Interventions for each group with sufficient detail to allow replication, including how and when they will be administered Page 7 Lines 6-10
	11b	Criteria for discontinuing or modifying allocated interventions for a given trial participant (eg, drug dose change in response to harms, participant request, or improving/worsening disease) Page 10 Line 28
	11c	Strategies to improve adherence to intervention protocols, and any procedures for monitoring adherence (eg, drug tablet return, laboratory tests) Page 10 Lines 23-27
	11d	Relevant concomitant care and interventions that are permitted or prohibited during the trial Page 10 Line 30 to Page 11 Line 1
Outcomes	12	Primary, secondary, and other outcomes, including the specific measurement variable (eg, systolic blood pressure), analysis metric (eg, change from baseline, final value, time to event), method of aggregation (eg, median, proportion), and time point for each outcome. Explanation of the clinical relevance of chosen efficacy and harm outcomes is strongly recommended Page 8
Participant timeline	13	Time schedule of enrolment, interventions (including any run-ins and washouts), assessments, and visits for participants. A schematic diagram is highly recommended (see Figure) Figures 1 and 2 and Supplementary Figure 1
Sample size	14	Estimated number of participants needed to achieve study objectives and how it was determined, including clinical and statistical assumptions supporting any sample size calculations Page 9 'Sample size' section
Recruitment	15	Strategies for achieving adequate participant enrolment to reach target sample size Page 13 Line 17 onwards

Methods: Assignment of interventions (for controlled trials)

**Allocation:**		
Sequence generation	16a	Method of generating the allocation sequence (eg, computer-generated random numbers), and list of any factors for stratification. To reduce predictability of a random sequence, details of any planned restriction (eg, blocking) should be provided in a separate document that is unavailable to those who enrol participants or assign interventions Page 9 Line 5
Allocation concealment mechanism	16b	Mechanism of implementing the allocation sequence (eg, central telephone; sequentially numbered, opaque, sealed envelopes), describing any steps to conceal the sequence until interventions are assigned Page 9 Line 7
Implementation	16c	Who will generate the allocation sequence, who will enrol participants, and who will assign participants to interventions Page 8 Line 29
Blinding (masking)	17a	Who will be blinded after assignment to interventions (eg, trial participants, care providers, outcome assessors, data analysts), and how Page 7 Line 2
	17b	If blinded, circumstances under which unblinding is permissible, and procedure for revealing a participant's allocated intervention during the trial
**Methods: Data collection, management, and analysis**		
Data collection methods	18a	Plans for assessment and collection of outcome, baseline, and other trial data, including any related processes to promote data quality (eg, duplicate measurements, training of assessors) and a description of study instruments (eg, questionnaires, laboratory tests) along with their reliability and validity, if known. Reference to where data collection forms can be found, if not in the protocol Trial schedule section Page 8 Line 28
	18b	Plans to promote participant retention and complete follow-up, including list of any outcome data to be collected for participants who discontinue or deviate from intervention protocols Page 10 Line 24
Data management	19	Plans for data entry, coding, security, and storage, including any related processes to promote data quality (eg, double data entry; range checks for data values). Reference to where details of data management procedures can be found, if not in the protocol
Statistical methods	20a	Statistical methods for analysing primary and secondary outcomes. Reference to where other details of the statistical analysis plan can be found, if not in the protocol Statistical Analysis Page 11 Line 15
	20b	Methods for any additional analyses (eg, subgroup and adjusted analyses) Page 12 Line 12

For peer review only - http://bmjopen.bmj.com/site/about/guidelines.xhtml

	20c	Definition of analysis population relating to protocol non-adherence (eg, as randomised analysis), and any statistical methods to handle missing data (eg, multiple imputation) Page 12 Line 1 onwards
**Methods: Monitoring**		
Data monitoring	21a	Composition of data monitoring committee (DMC); summary of its role and reporting structure; statement of whether it is independent from the sponsor and competing interests; and reference to where further details about its charter can be found, if not in the protocol. Alternatively, an explanation of why a DMC is not needed Supplementary data
	21b	Description of any interim analyses and stopping guidelines, including who will have access to these interim results and make the final decision to terminate the trial Page 10 Line 10
Harms	22	Plans for collecting, assessing, reporting, and managing solicited and spontaneously reported adverse events and other unintended effects of trial interventions or trial conduct Page 9 Line 13
Auditing	23	Frequency and procedures for auditing trial conduct, if any, and whether the process will be independent from investigators and the sponsor
**Ethics and dissemination**		
Research ethics approval	24	Plans for seeking research ethics committee/institutional review board (REC/IRB) approval Page 7 Line 4 and Page 12 Line 25
Protocol amendments	25	Plans for communicating important protocol modifications (eg, changes to eligibility criteria, outcomes, analyses) to relevant parties (eg, investigators, REC/IRBs, trial participants, trial registries, journals, regulators) Supplementary data
Consent or assent	26a	Who will obtain informed consent or assent from potential trial participants or authorised surrogates, and how (see Item 32) Page 9 Line 5
	26b	Additional consent provisions for collection and use of participant data and biological specimens in ancillary studies, if applicable Supplementary data (PIS and Informed Consent Form)
Confidentiality	27	How personal information about potential and enrolled participants will be collected, shared, and maintained in order to protect confidentiality before, during, and after the trial Supplementary data (PIS and Informed Consent Form)
Declaration of interests	28	Financial and other competing interests for principal investigators for the overall trial and each study site Page 20

review only

Access to data	29	Statement of who will have access to the final trial dataset, and disclosure of contractual agreements that limit such access for investigators
Ancillary and post-trial care	30	Provisions, if any, for ancillary and post-trial care, and for compensation to those who suffer harm from trial participation
Dissemination policy	31a	Plans for investigators and sponsor to communicate trial results to participants, healthcare professionals, the public, and other relevant groups (eg, via publication, reporting in results databases, or other data sharing arrangements), including any publication restrictions
	31b	Authorship eligibility guidelines and any intended use of professional writers Page 19
	31c	Plans, if any, for granting public access to the full protocol, participant-level dataset, and statistical code
**Appendices**		
Informed consent materials	32	Model consent form and other related documentation given to participants and authorised surrogates Supplementary data (PIS and Informed Consent Form)
Biological specimens	33	Plans for collection, laboratory evaluation, and storage of biological specimens for genetic or molecular analysis in the current trial and for future use in ancillary studies, if applicable Page 14 Line 1 onwards and Supplementary Figures 2 and 3

*It is strongly recommended that this checklist be read in conjunction with the SPIRIT 2013 Explanation & Elaboration for important clarification on the items. Amendments to the protocol should be tracked and dated. The SPIRIT checklist is copyrighted by the SPIRIT Group under the Creative Commons "Attribution-NonCommercial-NoDerivs 3.0 Unported" license.

For peer review only
